# Welfare economics of managing an epidemic: an exposition

**DOI:** 10.1007/s42973-021-00096-6

**Published:** 2021-10-18

**Authors:** Yasushi Iwamoto

**Affiliations:** Graduate School of Economics, University of Tokyo, Tokyo, Japan

**Keywords:** COVID-19, SIR model, Economic epidemiology, Cost–benefit analysis, Externalities, Lockdown, Value of a statistical life, D61, D62, E69, I18

## Abstract

This paper reviews recent findings on the normative analysis of private and governmental countermeasures against infectious diseases, focusing on COVID-19. Based on a model that relates the economic activity to infectious disease epidemics, policies that maximize social welfare are considered. Lockdowns in many countries are measures that restrict economic activity over a wide area, and the economic damage they cause is extremely large. Existing studies on the net benefit of lockdown implemented in 2020 have reached mixed conclusions as to whether it is warranted or not. Although the estimates of costs and effects are relatively stable, the setting of the value of a statistical life for converting effects into benefits has a wide range and is also likely to overestimate benefits. Therefore, a careful procedure for setting is particularly crucial to obtain a reliable evaluation of countermeasures. Compared to uniform restriction of activities, taking measures to restrict activities by selecting targets may improve efficiency. Attributes that can be used to select targets include those that can be identified at little or no cost, such as age and industry, and those that can only be identified at a cost, such as close contact with infectious individuals and the presence of pathogens. In comparison to lockdown, these measures may reduce human suffering and economic suffering. No trade-off exists between uniform activity restrictions and selective activity restrictions.

## Introduction

This paper reviews a framework for evaluating the benefits and costs of private economic agents’ prevention behavior and governmental non-pharmaceutical interventions against infectious diseases, particularly focusing on the economic analysis that has rapidly developed during the outbreak of COVID-19.

At the time of writing this article, Japan’s countermeasures against COVID-19 were able to contain several waves of the disease with a low death toll. The analysis and proposal of the Expert Committee have been influential in conducting the measures. However, epidemiology does not have models that consider the economic damages caused by prevention measures. If the policy advice is derived solely from such a model, it will have to ignore the economy.

The economic losses caused by COVID-19 and countermeasures against it were huge, surpassing the swine flu epidemic that began in 1918. Moreover, it is deemed to be the biggest economic shock since the Great Depression. In policy responses to financial crises and natural disasters, we consider mitigating unwanted shocks. However, war and epidemics are different from other economic shocks in that they voluntarily sacrifice the economy for other purposes. When the survival of a country is at stake in a war, there may be no room for economic considerations. However, in the case of COVID-19, the trade-off between health and economy becomes a serious concern for policymakers.

Even if economists are faithful to their professions, they will not propose a solution that neglects health. Economics sets individual preferences as a criterion for policy evaluation; thus, if an individual values both health and economy, then both will be considered in policy evaluation criteria. Then, we can consider the advantageous choice in the trade-off between health and economy with the model, which can describe the relationship between infectious disease epidemics and economic activities. This paper aims to explain such a framework.

This paper summarizes the findings of recent studies that expressed the trade-off between health and economy by combining the simplest possible macroeconomic model and the SIR (susceptible–infected–recovered) model (Kermack & McKendrick, 1927) that describes infectious disease epidemics in epidemiology. This paper discusses the relationship between epidemics and economy through the following two pathways. First, in epidemiological models, the spread of infectious disease is related to the contact opportunities of people,^1^ and by relating these contact opportunities to economic activities, we can also link the epidemic of infectious diseases and economic activities.^2^ Second, from the dynamic optimization problem of a representative individual, we derive that the impact of infectious diseases and countermeasures against it includes the impact of economic and fatality losses. Thus, the countermeasure is described as a policy that restrains the economic activity from deterring the spread and reducing the impacts on the health of population.

This paper focuses on how to design desirable countermeasures against infectious diseases from the viewpoint of the trade-off between health and economy. It also builds a model that can express this viewpoint and presents the selected findings of recent research. Therefore, the research described here is limited to a small portion of the economic analysis that has appeared surprisingly in a short period since the emergence of COVID-19.^3^^,^^4^

Even within this narrow focus, several simplifying assumptions are made to avoid undue complexity. One of the most important assumptions is the use of representative individuals to evaluate welfare. This ignores the differences in instantaneous utility between infectious and non-infectious individuals, and the fact that in reality, economic shocks are skewed toward the vulnerable. The model implicitly assumed that income redistribution policies are implemented to smooth these heterogeneous burdens. In other words, the infectious disease control measures discussed in this paper implicitly include such income redistribution policies.

Regarding the trade-off between health and economy in infectious disease control, the qualitative structure of the model has been well established. However, even though research has progressed rapidly, quantitative implications have not yet been stabilized. The present paper reviews the two streams of studies on lockdown conducted in 2021. The first is an early attempt of simulation analysis that inquires the optimal form of lockdown. Many parameters cannot be based on actual experiences; hence, determining the appropriate number of weeks for lockdown is difficult. We focus on the qualitative implications. Some simulation analysis results will also be presented, but quantitative information should not be interpreted as definitive. However, it should be viewed with a wide margin of error. The second is ex post cost–benefit analyses, which do not have to pinpoint the optimal policy but ask whether the actual actions brought a net benefit to the society. Although their parameter setting can utilize actual experiences, some parameters have a wide range among studies, thereby producing mixed results on welfare consequences. Particularly, the monetization of effect (reduction of death toll) is far from stabilized.

The first type of infectious disease countermeasure to be examined is a lockdown or stay-at-home order that uniformly restricts economic activity. Next, we examine the cost-effectiveness of measures that are not uniform and are targeted at specific groups. The policies examined here are those that aim to isolate infected people through testing, protect high-risk populations, and impose industry-specific restrictions. Comparisons of measures with widely varying costs have yielded somewhat robust results, even with uncertainties in quantifying benefits and costs. This means that rather than uniformly restricting economic activity to control the infection, focusing on the most effective targets and applying restrictions are preferred. The remainder of this article is organized as follows to demonstrate this point.

Section 2 presents an SIR model that describes infectious disease epidemics, but does not specify economic activity. Section 3 starts with a macroeconomic model that does not consider infectious disease and formulates the relation between economic activity and infectious disease. Section 4 explains the analytical framework needed to analyze the welfare of countermeasures. Section 5 discusses the lockdown properties as a solution to the optimization problem and reviews some earlier simulation results related to the lockdown against the first wave of COVID-19. Section 6 reviews ex post evaluations of lockdown in 2020.

Sections 7 and 8 examine some infection control targeting specific populations. In particular, Sect. 7 examines two types of testing and isolation strategies: uniform testing and contact tracing. Uniform testing is more efficient than lockdown, whereas contact tracing, which is a kind of targeted testing, is more efficient than uniform testing. Section 8 considers measures to impose activity restrictions based on observable attributes without testing. To represent such measures, we use a multidimensional SIR model describing a society that is composed of heterogeneous individuals. The measures discussed here are those that protect high-risk elderly people and do not restrict the activities of younger people, and those that impose different activity restrictions based on the risk of each industry.

## Modeling epidemic

### Simple SIR model

We set up a continuous-time SIR model. Agents in the economy consist of susceptible (*S*), infectious (*I*), and removed (recovered with immunity or dead; *R*). The population at the initial point (time 0) is normalized as 1. Then,

$$S\left(0\right)+I\left(0\right)=1,$$

$$R\left(0\right)=0.$$

No infectious population exists before time 0, and a small number of infectious people enter at time 0, and an epidemic begins. Assuming no new entrance at time *t* (from the initial point to the infinite future) yields

1 $$\begin{array}{c}S\left(t\right)+I\left(t\right)+R\left(t\right)=1.\end{array}$$

The cumulative infectious is $1-S$. Meanwhile, population (living people) is denoted by *N*, and the cumulative death is $1-N$.

Newly infectious agent (the decrease in susceptible) results from a contact between the susceptible and the infectious, and is represented by

2 $$\begin{array}{c}{\text{New}}\left(t\right)=S\left(t\right)C\left(t\right)p\left(t\right)\frac{I\left(t\right)}{N\left(t\right)},\end{array}$$

where *C* is the contact rate (the number of people whom one person contacts in a unit time), *p* is the secondary attack rate (the ratio of newly infectious to people whom an infectious contacts with), and $I/N$ is the prevalence rate of community (the ratio of infectious to total population). Begon et al. (2002) contrasted the two specifications of the contact rate. One is “density-dependent” contact, where the contact rate is proportional to the population density ($C\propto N$ in the constant area).^5^ The other is “frequency dependent”, where the contact rate is independent of other variables. The new infection under the density-dependent contact becomes

3 $$\begin{array}{c}{\text{New}}\left(t\right)=\beta \left(t\right)S\left(t\right)I\left(t\right).\end{array}$$

Meanwhile, the new infection under the frequency-dependent contact is

4 $$\begin{array}{c}{\text{New}}\left(t\right)=\beta \left(t\right)S\left(t\right)\frac{I\left(t\right)}{N\left(t\right)},\end{array}$$

where *β* is the transmission rate. Under the density-dependent contact, when the population becomes double, the new infection becomes quadruple (quadratic homogeneity). Under the frequency-dependent contact, when the population becomes double, the new infection becomes double (linear homogeneity).

The modeling of infection has some similarities with the matching function in search theory in economics.^6^ For example, the matching function between the employed (*U*) and vacancies (*V*) in the labor market represented by ${U}^{{\alpha }_{1}}{V}^{{\alpha }_{2}}$, where ${\alpha }_{1}={\alpha }_{2}=1$ (quadratic matching) is second-order homogeneous, which is the same as density-dependent contact. When ${\alpha }_{1}+{\alpha }_{2}=1$, the matching function is linear homogenous and is similar to frequency-dependent contact. In the following, the formulation is based on Eq. (3). This choice is rationalized either under the setting of density-dependent contact or by assuming that the change in population (due to death) in Eq. (4) is small enough to be ignored.

The new infectious agent comes from susceptible agent. The change in the number of susceptible is

$$-\dot{S}\left(t\right)=\mathrm{New}\left(t\right).$$

Moreover, the infectious recover from the disease or die at the probability γ. The change in the number of infectious is

5 $$\begin{array}{c}\dot{I}\left(t\right)={\text{New}}\left(t\right)-\gamma I\left(t\right).\end{array}$$

The infectious period (average period of being infectious) is $1/\gamma$, and the effective reproduction $\mathcal{R}$ is defined as

6 $$\begin{array}{c}R\left(t\right)\equiv \frac{1}{\gamma }\frac{\mathrm{New}\left(t\right)}{I\left(t\right)},\end{array}$$

which represents how many persons one infectious person infects per infectious period. Rearranging (6) and substituting it into (5) yield

$$\dot{I}\left(t\right)=\left(\mathcal{R}\left(t\right)-1\right)\gamma I\left(t\right).$$

When the effective reproduction number is greater than 1, the disease will spread. Meanwhile, if the effective reproduction number is less than 1, the epidemic will eventually end.

A simple SIR model often assumes the transmission rate *β* is a constant. This implies that the behavior of people and government is not changed by the epidemic. This constant is denoted by ${\beta }_{0}$. Basic reproduction number is defined as ${\mathcal{R}}_{0}\equiv {\beta }_{0}/\gamma$. This is also an effective reproduction number in the pre-epidemic state, where total population is susceptible. The change in the number of infectious people is

7 $$\begin{array}{c}\dot{I}\left(t\right)=\left({\mathcal{R}}_{0}S\left(t\right)-1\right)\gamma I\left(t\right).\end{array}$$

Meanwhile, new death (a decrease in population) is given as

8 $$\begin{array}{c}-\dot{N}\left(t\right)=\phi \left(I\left(t\right)\right)\gamma I\left(t\right).\end{array}$$

Due to the limit of medical resources, the fatality rate $\phi$ is assumed to be higher when the number of patients is large (${\phi }^{^{\prime}}>0$). When each agent does not consider this impact, this setting generates the externality which government intervention addresses. The model is called an susceptible-infected-recovered-deceased (SIRD) model because containing the fatality is a necessary element to count the human damage. Since we describe the model as close to an SIR model as possible, we conveniently call it the SIR model.

### Evolution of effective reproduction number

The effective reproduction number decreases with time for several reasons. First, even if the transmission rate remains constant as the infection spreads, the effective reproduction number declines as the size of susceptible people shrinks because $\mathcal{R}\left(t\right)={\mathcal{R}}_{0}S\left(t\right)$ (Eq. 7). This is because the new infection decreases as the number of immunized people (recovered people in the model) increases around the infectious people. When the proportion of infectious people is greater than the reciprocal of the (fixed) basic reproduction number, the epidemic begins to converge. This is the formation of herd immunity.

The effective reproduction number also decreases as the transmission rate decreases due to the following factors.

One factor is heterogeneity. When the risk of infection varies from person to person, the average speed of infection decreases; hence, the effective reproduction number decreases. This is because those who are more likely to be infected first become infected, and those at the lower risk of infection remain among the susceptible, thus lowering the average rate of infection among the susceptible. The establishment of herd immunity is determined based on this lowered transmission rate; thus, herd immunity will form faster than predicted, assuming that the basic reproduction number is invariant.^7^

Although herd immunity and heterogeneity are mechanisms by which the effective reproduction number will decrease without behavioral changes, the effective reproduction number can also decrease when the private sector and the government take countermeasures against infectious disease. First, individuals and firms may voluntarily take disease prevention measures. Second, the government may encourage behavioral change beyond voluntary prevention or impose stronger measures, such as restrictions on behavior (e.g., lockdown and stay-at-home order).^8^

Although relatively inexpensive behavioral changes may sustain permanently, many other behavioral changes and restrictions involve economic and psychological costs. They should be used as temporary measures against infectious diseases that cannot be sustained for a long time. Therefore, whether herd immunity is achieved is not crucial in many infectious disease control measures. If the virus cannot be eradicated, much of the behavioral change resulting from policies will be used to “buy time” by temporarily deterring the spread of infection. We will discuss the benefits of buying time in the following.

## Macroeconomics of infectious disease

### Pre-epidemic economy

Suppose that before an epidemic emerges, individuals were homogenous and susceptible, and the economy was at the level of potential output. The SIR model usually describes epidemics over a short period; therefore, we do not consider investment and assume income to equal consumption.^9^ Although the utility is derived from consumption, we will describe it as a function of income. Social welfare is the simple sum of individual utility and is given by

$${V}_{0}\equiv {\int }_{0}^{\infty }{\mathrm{e}}^{-\rho t}{N}_{0}u\left(\overline{Y }\right)\mathrm{d}t=\frac{{N}_{0}u(\overline{Y })}{\rho },$$

where *ρ* is the discount rate and $\overline{Y }$ is the potential output in terms of per capita. In the pre-epidemic economy, nobody dies, and population is constant and normalized by 1, so that ${N}_{0}=1$. For a variable *x*, ${x}_{0}$ is the value of pre-epidemic economy (the steady state without an infectious disease), and $x\left(0\right)$ is the initial value on the epidemic path.

### Economy without countermeasures

Next, we consider an economy without any countermeasures against infectious diseases. The SIR model, which assumes that the transmission rate ${\beta }_{0}$ will be unchanged after the onset of the epidemic, cannot be used to predict a spread of the epidemic because people will act to prevent infection to protect themselves even in the absence of government interventions. Such an assumption is used as a counterfactual to analyze the effectiveness of countermeasures against infectious diseases. In other words, they are used to estimate the suffering from infectious diseases.

Two types of major damages are suffered from infectious diseases: health-related loss caused by deaths and economic losses caused by the inability to work due to the worsening health of infected people. Suppose that a specific portion of the infectious will be unable to work and that production will decline by that percentage. If this loss is spread uniformly across the entire economy through insurance or income redistribution policy, its impact on the economy is not very large. The production due to the absence of infected people may be disregarded in the analysis of managing an epidemic.^10^ In addition, although a decline in “quality of life” due to deteriorating health is also a factor in the decline in utility, many studies do not take it into account.

### The value of vaccine

When the vaccine is not developed, social welfare is defined by the simple sum of individual utilities as

$${\int }_{0}^{\infty }{\mathrm{e}}^{-\rho t}N\left(t\right)u\left(Y\left(t\right)\right)\mathrm{d}t.$$

This setting of social welfare function implicitly assumes that the intra-temporal insurance perfectly smoothens any related losses due to the epidemic; thus, no cross-sectional difference exists in individual income. Although this assumption ignores heterogeneous burdens observed in the real world, it attempts to capture the desirable, partially implemented policy responses that offset the welfare loss due to uneven burdens. The social welfare function aggregates instantaneous individual utility at each time and then integrates it over time.

The value of vaccine is typically incorporated in the early studies of managing an epidemic as follows. When the vaccine is developed at time *t*, the epidemic is assumed to become not severe, and the economy returns to the pre-epidemic state. In this setting, the social welfare is defined as

9 $$\int_{0}^{t} {{\text{e}}^{{ - \rho s}} N(s)u(Y(s)){\text{d}}s + } \int_{t}^{\infty } {{\text{e}}^{{ - \rho s}} N(t)u(\bar{Y}){\text{d}}s} .$$

The first term of (9) is the social welfare when the infectious diseases prevail until time *t*, and the second term is that after the economy returns to the pre-epidemic economy.

The period of the vaccine availability is not known ex ante; thus, the timing of arrival is stochastically set hereafter. Assume that the arrival of vaccine follows a Poisson process with a probability *ν* at each time. The probability that the vaccine is available until time *t* is given by

$$F\left(t\right)=1-{\mathrm{e}}^{-\nu t}.$$

Moreover, the probability of arriving *t* is

$$\dot{F}\left(t\right)=\nu {\mathrm{e}}^{-\nu t}=\nu \left(1-F\left(t\right)\right).$$

The lockdown plays an important role in an early epidemic phase when practical issues related to vaccines are not serious yet. The above settings of vaccine availability are useful for the analysis of lockdown because they keep the dynamics not distant from the no-vaccine case. However, this strategic ignorance of vaccine-related issues pays some costs by ignoring some important policy issues. First, the entire population cannot be vaccinated instantaneously. Since the setting of stochastic arrival can be interpreted as the deterministic gradual vaccination, the stochastic arrival model can partially depict the pattern of actual vaccination. Second, although the expected waiting period before the vaccine’s arrival is constant at each moment under the Poisson process, the actual arrival of the vaccine may become predictable as vaccine trials progress. This predictable arrival may create an intriguing externality, as will be discussed in Sect. 5.2.

Using the integration-by-parts formula, we can rewrite the expected social welfare as

10 $$\begin{aligned} V\left( 0 \right) & = \mathop \int \limits_{0}^{\infty } \dot{F}\left( t \right)\left[ {\mathop \int \limits_{0}^{t} {\text{e}}^{{ - \rho s}} N\left( s \right)u\left( {Y\left( s \right)} \right){\text{d}}s + \mathop \int \limits_{t}^{\infty } {\text{e}}^{{ - \rho s}} N\left( t \right)u\left( {\bar{Y}} \right){\text{d}}s} \right]{\text{d}}t \\ & = \mathop \int \limits_{0}^{\infty } {\text{e}}^{{ - \left( {\rho + \nu } \right)t}} N\left( t \right)u\left( {Y\left( t \right)} \right){\text{d}}t + \mathop \int \limits_{0}^{\infty } {\text{e}}^{{ - \rho t}} N\left( 0 \right)u\left( {\bar{Y}} \right){\text{d}}t \\ & \quad - \mathop \int \limits_{0}^{\infty } {\text{e}}^{{ - \left( {\rho + \nu } \right)t}} N\left( t \right)u\left( {\bar{Y}} \right){\text{d}}t + \mathop \int \limits_{0}^{\infty } \left[ {\dot{N}\left( t \right){\text{e}}^{{ - \nu t}} \mathop \int \limits_{t}^{\infty } {\text{e}}^{{ - \rho s}} u\left( {\bar{Y}} \right){\text{d}}s} \right]{\text{d}}t \\ & = V_{0} - \mathop \int \limits_{0}^{\infty } {\text{e}}^{{ - \left( {\rho + \nu } \right)t}} N\left( t \right)\left[ {u\left( {\bar{Y}} \right) - u\left( {Y\left( t \right)} \right)} \right]{\text{d}}t \\ & \quad - \mathop \int \limits_{0}^{\infty } \left[ {{\text{e}}^{{ - \left( {\rho + \nu } \right)t}} \left( { - \dot{N}\left( t \right)} \right)\mathop \int \limits_{t}^{\infty } {\text{e}}^{{ - \rho \left( {s - t} \right)}} u\left( {\bar{Y}} \right){\text{d}}s} \right]{\text{d}}t. \\ \end{aligned}$$

Defining the negative of output gap, or economic loss (the ratio to potential output) as

$$y\left(t\right)\equiv -\frac{Y\left(t\right)-\overline{Y} }{\overline{Y} },$$

we can write the output as

11 $$\begin{array}{c}Y\left(t\right)=\left(1-y\left(t\right)\right)\overline{Y }.\end{array}$$

Rearranging (11) yields

$$-y\left(t\right)=\frac{Y\left(t\right)-\overline{Y} }{\overline{Y} },$$

where *y* is the negative of output gap, or economic loss (the ratio to potential output).

Rearranging (10) and substituting (8) and (11) in it yields another representation of the welfare loss from the pre-epidemic economy:

12 $$\begin{array}{c}{V}_{0}-V\left(0\right)={\int }_{0}^{\infty }{\mathrm{e}}^{-\left(\rho +\nu \right)t}\left\{N\left(t\right)\left[u\left(\overline{Y }\right)-u\left(\left(1-y\left(t\right)\right)\overline{Y }\right)\right]+\phi \left(I\left(t\right)\right)\gamma I\left(t\right){\lambda }_{N,0}\right\}\mathrm{d}t,\end{array}$$

where the lifetime utility at time *t* in the pre-epidemic economy is

$${\lambda }_{N,0}\equiv {\int }_{0}^{\infty }{\mathrm{e}}^{-\rho t}u\left(\overline{Y }\right)\mathrm{d}t={\int }_{t}^{\infty }{\mathrm{e}}^{-\rho \left(s-t\right)}u\left(\overline{Y }\right){\text d}s,$$

which actually does not depend on *t*. The welfare loss consists of two parts: one is the loss of income caused by the countermeasures to the epidemic, and the other is the lost utility of the dead.

## Framework of welfare analysis

### Necessities of government interventions

In evaluating non-pharmaceutical intervention (NPI), we must consider that economic agents also voluntarily take infection prevention measures. When we solve the problem of maximizing the social welfare function, one considers measures for the private and government sectors together. If social benefits and costs are properly reflected in individuals’ incentives, NPI need not be added beyond private prevention measures. Therefore, if NPI is needed, a deviation exists between the private and social benefits. Even if economic agents are rational, two kinds of externalities are highlighted in the literature.

The first kind of externality is that in the case of density-dependent contact infection as in Eq. (3), for example, if an individual refrains from going out by a ratio of *z*, the number of contacts decreases to $\left(1-z\right)C$, given the behavior of others. However, if everyone refrains from going out only in the proportion of *z*, the number of contacts when going out will decrease by the proportion of $1-z$, and the total number of opportunities for infection can be expected to decrease by ${\left(1-z\right)}^{2}$. The social marginal benefit of self-restraint is twice as large as the private marginal benefit.^11^ Garibaldi et al. (2020b) and Gonzalez-Eiras and Niepelt (2020b) highlighted the second kind of externality; that is, individuals do not recognize the effect of prevention behavior on the aggregate number of infectious, which is a state variable of the SIR model. We will discuss the properties of this externality in Sect. 5.2, where the dynamics of state variables are elaborated.

If individuals are not rational or their information is not perfect, the government may correct the behavioral bias of private agents. For example, it may be effective for the government to provide information on unknown infectious diseases because they do not have sufficient knowledge beforehand. Nudges may also encourage private actors to take prevention measures.

The measures to resolve externalities include direct regulation and Pigouvian taxes and subsidies. The externality that arises here is that businesses will operate without bearing the cost of the outbreak. This creates a gap between the private benefits of the business and the social benefits. If we choose to use the Pigou tax/subsidy instrument, we have the option of giving compensation to the operators for stopping the event or imposing a penalty for operating. Coase’s theorem states that if the business is initially granted the right to operate with the risk of spreading the infection, it should be compensated for refraining from operating, and if it is not initially granted the right to operate, it should be fined for operating. If the level of compensation and fines is appropriate (and no transaction costs are involved), an efficient allocation of resources can be achieved in any case. However, the income earned by the business depends on the initial allocation of property rights. If the self-restraint lasts for a long time, the business faces difficulty operating without compensation. Moreover, if the situation worsens further, there will be welfare losses from the liquidation of business assets.

Thus, the allocation of property rights has an important impact on the design of infection prevention and control. The operations of smooth economic activities need to ensure that property rights are clarified in advance to enhance the predictability of business activities. Discussing whether or not to compensate restrictions on economic activities after an epidemic would shake the very basis of economic development, which is the establishment of property rights. In addition, it would have a more negative impact on economic activities than the impact of restrictions on economic activities.

### Measures of NPI

In order of increasing cost, the instruments of NPI include nudges, social distancing, universal mask wearing, testing and isolation, and lockdown. Social distance is conducted voluntarily mainly by the private sector, but the government can also enforce it. In real life, a huge difference exists between social distancing, where people are free to go out, and lockdown, where people are restricted from going out. However, distinguishing them is hard in the abstract model, which sees only the effect of restrictions on the occurrences of infection. In fact, the structure of the model is the same, but depending on the paper, it is called social distancing or lockdown. Practically, social distancing and lockdown are roughly distinguished by the amount of cost.

Although lockdown is a means of imposing uniform restrictions (except for essential workers), testing and isolation are a means of imposing restrictions on targeted groups. Since the size of the groups subject to the restrictions is much smaller than the total population, the economic loss due to restrictions on behavior is an order of magnitude smaller than that of lockdown. What needs to be considered is the need for costs of tracking contacts and how much the effect is weakened by limiting the target. This point will be discussed in Sect. 7 onwards.

### Value of a statistical life

The trade-off between health and economy is a key concern in managing COIVD-19. Unlike the pre-epidemic economy, the output after the introduction of epidemic may fluctuate. The functional form of the social welfare function can also represent the expected lifetime utility of the representative agent, where *N* denotes the surviving probability of the representative agent. Here, we take this setting. The expected lifetime utility of representative agent is defined as

13 $$\begin{array}{c}{\lambda }_{N}\left(0\right)\equiv \int_{0}^{\infty }{\mathrm{e}}^{-\rho t}N\left(t\right)u\left(Y\left(t\right)\right)\mathrm{d}t,\end{array}$$

where $$N\left(0\right)=1$$.^12^

The value of a statistical life, formulated originally by Schelling (1968), plays an important role in framing the following analysis. It is defined as the willingness to pay (WTP; how much they can sacrifice the consumption at the initial point) for a small increase in surviving probability. In this model, it is defined as the marginal rate of substitution of $N\left(0\right)$ for $Y\left(0\right)$. Totally differentiating (13) yields the change in lifetime utility due to the changes in the initial values of *N* and *Y*:

$$\mathrm{d}{\lambda }_{N}\left(0\right)={\lambda }_{N}\left(0\right)\mathrm{d}N\left(0\right)+{u}^{\prime}\left(Y\left(0\right)\right)\mathrm{d}Y\left(0\right).$$

The value of a statistical life is then written as

14 $$- \left. {\frac{{{\text{d}}Y(0)}}{{{\text{d}}N(0)}}} \right|_{{{\text{d}}\lambda _{N} \left( 0 \right) = 0}} = \frac{{\lambda _{N} (0)}}{{u^{\prime}(Y(0))}},$$

where the lifetime utility is monetized by marginal utility of consumption.^13^

When consumption is constant in (13), we obtain a more operative formula for the value of a statistical life from (14) as

15 $$\begin{array}{c}\frac{u\left(Y\right)}{{u}^{\prime}\left(Y\right)}{\int }_{0}^{\infty }{\mathrm{e}}^{-\rho t}N\left(t\right)\mathrm{d}t\equiv \frac{u\left(Y\right)}{{u}^{\prime}\left(Y\right)}\text{LE}\left(\rho \right),\end{array}$$

where $\mathrm{LE}\left(\rho \right)$ is the discounted life years.

The value of a statistical life has been estimated from the relationship between mortality risk and wage differentials between industries, in line with the concept of compensating wages. It is also estimated by asking the WTP for measures to reduce mortality risk using contingent valuation methods. Estimates from these studies have been used in practice to evaluate safety-related regulations and policies. Similarly, Glover et al. (2020), Gollier and Straub (2020) and Scherbina (2020), who assessed lockdowns, used the estimates along these lines. However, Hall et al. (2020) and Pindyck (2020) revealed that the risk of death from COVID-19 is much greater than the risk assumed by the studies estimating the value of a statistical life, and that using conventional estimates would lead to exaggerated estimates.

Based on (15), the WTP to finite increase in surviving probability is defined as follows. Suppose a decrease in consumption, *α*, offsets the utility gain from lengthening life expectancy by the proportion of *δ*. Then, *α* satisfies

16 $$\begin{array}{c}u\left(Y\right)-u\left(Y-\alpha \right)=u\left(Y\right)\left(1+\delta \right)\text{LE}\left(\rho \right)-u\left(Y\right)\text{LE}\left(\rho \right).\end{array}$$

Linear approximation of left-hand side of (14) yields

$${u}^{\prime}\left(Y\right)\alpha \approx u\left(Y\right)\delta \mathrm{LE}\left(\rho \right),$$

which gives the value of a statistical life based on (13). However, the larger *δ* is, the larger the error of Taylor approximation is.

Hall et al. (2020) provided the following numerical example. According to the United States Environmental Protection Agency’s (EPA) setting, the value of 1 year of life expectancy amounts to 6 years of consumption. Since the deaths are skewed toward the elderly, the life expectancy of the deceased is calculated to be 14.5 years, and the value of a statistical life of the deceased is calculated to be 87 years of consumption. Ferguson et al. (2020) estimated that the fatality rate (death/case) is 0.81%. Then, avoiding a fatality rate of 0.81% (eliminating infectious diseases) is calculated to be worth 70.5% of annual consumption (0.81% × 14.5 × 6).

In contrast, based on Eq. (16), the same gain was estimated to be 41.3% of annual consumption under their favorite functional form of utility. This shows that the linear approximation overstates the value of a statistical life by 70%. Also, an increase in life expectancy of 1 year is worth 3.5 years of consumption. Note that these numbers will vary depending on the specification of the utility function.

Alvarez et al. (2020), who studied the timing and scale of the optimal lockdown, set the growth in life expectancy of 1 year to be equivalent to 3 years of consumption, in line with the estimates of Hall et al. (2020). Since the life expectancy of the deceased is assumed to be 10 years, the value of a statistical life of the deceased is about two-thirds of that in Hall et al. (2020). In addition, the ratio of consumption to GDP is taken as two-thirds so that it can be compared with the loss of economic activity expressed as a percentage of GDP. Hence, the value of a statistical life of deaths was set to be equivalent to 20 years of GDP per capita. Sensitivity analysis was also conducted in which the statistical life value was changed to 10, 30, 80, and 140 years of GDP per capita. The last 140 years of GDP is roughly equivalent to 200 years of consumption, which is the aforementioned EPA's set value. Table 1 summarizes the EPA’s setting, the estimates of Hall et al. (2020), and the reference case of Alvarez et al. (2020).

**Table 1:** The value of a statistical life

	Value of a living year	Average remaining life of dead	Value of a statistical life
EPA	6 years of consumption	40 years	240 years of consumption
Hall et al.	3.5 years of consumption	14.5 years	51 years of consumption
Alvarez et al.	3 years of consumption	10 years	30 years of consumption, or 20 years of GDP

Consumption and GDP are per capita

Since the magnitude of the risk from COVID-19 is far greater than the risk focused on by conventional estimates of the value of a statistical life, obtaining reasonable estimates from people’s actual choices is difficult. Given the uncertainty of the value of a statistical life, the sensitivity analysis would be particularly valuable. Acemoglu et al. (2020a) depicted the health and economic consequences of optimal policies under alternative settings of the value of a statistical life in a two-dimensional graph, as another method of dealing with the uncertainty. The trajectory of optima represents the efficiency frontier under the trade-off between health and economy.

## Lockdown

### Benefit and cost of managing epidemic

To make the framework of cost–benefit analysis applicable, we consider the monetized welfare loss. Taylor expansion of per capita potential output in the right-hand side of (12) is

$$u\left(\left(1-y\left(t\right)\right)\overline{Y }\right)=u\left(\overline{Y }\right)+{u}^{\prime}\left(\overline{Y }\right)\left(\left(1-y\left(t\right)\right)\overline{Y }-\overline{Y }\right).$$

The utility loss from the potential GDP is represented by

17 $$\begin{array}{c}u\left(\overline{Y }\right)-u\left(\left(1-y\left(t\right)\right)\overline{Y }\right)\approx {u}^{\prime}\left(\overline{Y }\right)y\left(t\right)\overline{Y }.\end{array}$$

Meanwhile, the value of a statistical life evaluated at the pre-epidemic economy, VSL, is defined by

18 $$\begin{array}{c}\text{VSL}\equiv \frac{{\lambda }_{N,0}}{{u}^{\prime}\left(\overline{Y }\right)}.\end{array}$$

Substituting (17) and (18) into (12) and rearranging it yield

$$\frac{{V}_{0}-V\left(0\right)}{{u}^{\prime}\left(\overline{Y }\right)}\approx {\int }_{0}^{\infty }{\mathrm{e}}^{-\left(\rho +\nu \right)t}\left[N\left(t\right)y\left(t\right)\overline{Y }+\phi \left(I\left(t\right)\right)\gamma I\left(t\right)\mathrm{VSL}\right]{\text d}t,$$

which indicates the monetary value of welfare loss. The first term in the bracket of the right-hand side is economic loss represented by the output gap, and the second term is human loss, which is the product of the number of death and the value of statistical life.

The desirable countermeasure to the infectious disease is obtained by solving the problem of minimizing the monetized welfare loss, which is given by

19 $$\begin{array}{c}\underset{y\left(t\right)}{\mathrm{min}}{\int }_{0}^{\infty }{\mathrm{e}}^{-\left(\rho +\nu \right)t}\left[N\left(t\right)y\left(t\right)\overline{Y }+\phi \left(I\left(t\right)\right)\gamma I\left(t\right)\mathrm{VSL}\right]{\text d}t.\end{array}$$

The control variable is represented by *y*, which may be determined by practical measures, say *z*, like the refrained going out ratio in Sect. 4.1. Controlling *y* is equivalent to choosing *z* and setting $y=y\left(z\right)$. Eliminating *S* from (3) by using (1) and employing a flexible functional form yield the function that drives new infections:

20 $$\begin{array}{c}{\text{New}}\left(1-y\left(t\right),I\left(t\right),R\left(t\right)\right).\end{array}$$

Equation (20) implies that the new infection is affected by *y* because the transmission rate depends on the contact rate, which in turn is a function of economic activity. This equation relates the prevalence of infectious disease to economic activity. The objective function contains the economic activity and human loss due to infectious disease. These relationships characterize the trade-off between health and economy.

Substituting (20) into (5) yields

21 $$\begin{array}{c}\dot{I}\left(t\right)={\text{New}}\left(1-y\left(t\right),I\left(t\right),R\left(t\right)\right)-\gamma I\left(t\right).\end{array}$$

The dynamics of the removed is given by

22 $$\begin{array}{c}\dot{R}\left(t\right)=\gamma I\left(t\right).\end{array}$$

From (8), the population dynamics becomes

23 $$\begin{array}{c}\dot{N}\left(t\right)=-\phi \left(I\left(t\right)\right)\gamma I\left(t\right).\end{array}$$

The desirable policy is obtained by solving the problem of minimizing (19) subject to the equations of three state variables: (21), (22), and (23). Define the Hamiltonian as

$${H}^{*}\left(t\right)={\mathrm{e}}^{-\left(\rho +\nu \right)t}H\left(t\right)={\mathrm{e}}^{-\left(\rho +\nu \right)t}\left\{N\left(t\right)y\left(t\right)\overline{Y }+\phi \left(I\left(t\right)\right)\gamma I\left(t\right)\mathrm{VSL}+{\lambda }_{I}\left(t\right)\left[\mathrm{New}\left(1-y\left(t\right),I\left(t\right),R\left(t\right)\right)-\gamma I\left(t\right)\right]+{\lambda }_{R}\left(t\right)\gamma I\left(t\right)+{\lambda }_{N}\left(t\right)\left[-\phi \left(I\left(t\right)\right)\gamma I\left(t\right)\right]\right\}.$$

The co-state variables, ${\lambda }_{I}$, ${\lambda }_{R}$, and ${\lambda }_{N}$, are the Lagrangian multiplier of each state variable. If the optimization problem has an interior solution,^14^ the condition for the optimal countermeasure is given as

24 $$\begin{array}{c}\frac{\partial H\left(t\right)}{\partial y\left(t\right)}=N\left(t\right)\overline{Y }-{\lambda }_{I}\left(t\right)\frac{\partial \mathrm{New}\left(t\right)}{\partial \left(1-y\left(t\right)\right)}=0.\end{array}$$

As explained in Sect. 4, the infection prevention and control measures considered here are a combination of voluntary prevention behavior and NPI. Although lockdown aims to restrict economic activity in combination with voluntary prevention behavior, focusing on the restraints of economic activity in this form is useful to know where the interventions should go. To understand the part where NPI restrains economic activity, we need to solve the dynamic optimization problem of the private sector when the government does not intervene. We also need to find the restraint of economic activity by voluntary prevention behavior and take the difference from the restraint of economic activity in the society as a whole. However, we will not enter into this discussion in this paper, which does not explicitly model the optimizing behavior of the private sector.

Let us consider the implications of the first-order condition. Rearranging (24) yields

25 $$\begin{array}{c}{\lambda }_{I}\left(t\right)\frac{\partial \mathrm{New}\left(t\right)}{\partial \left(1-y\left(t\right)\right)}=N\left(t\right)\overline{Y },\end{array}$$

which shows that the marginal benefit of restricting economic activities (left-hand side) equals the marginal cost (right-hand side). The cost is the loss of output measured as the decline of the output gap. Meanwhile, the benefit is calculated as the effect of lowering economic activities on reduced new infectious ($\partial \mathrm{New}\left(t\right)/\partial \left(1-y\left(t\right)\right)$) multiplied by the positive welfare effect of lowering the new infection (${\lambda }_{I}\left(t\right)$). An alternative interpretation of ${\lambda }_{I}$ is that it represents the loss caused by an infectious.

To evaluate the benefits, we need to determine three things: (1) the value of a statistical life, (2) the projection of the number of infected people and the fatality rate, and (3) the relationship between economic activity and the infection rate. The projection of the number of infected people and the fatality rate are essential to know the number of deaths that will occur from newly infected people (including the effects of second and subsequent infected people and those who recover).

Ferguson et al. (2006) assumed that 30% of influenza virus infections in the USA occur in the home, 37% in schools and workplaces, and 33% in the community. This assumption was also used in the simulation of the COVID-19 epidemic by Ferguson et al. (2020). An example of decomposing (2) by the situation of contact is written as

$$\mathrm{New}\left(t\right)=S\left(t\right)\left[{C}^{\mathrm{home}}\left(t\right){p}^{\mathrm{home}}\left(t\right)+{C}^{\mathrm{school}}\left(t\right){p}^{\mathrm{school}}\left(t\right)+{C}^{\mathrm{work}}\left(t\right){p}^{\mathrm{work}}\left(t\right){+C}^{\mathrm{others}}\left(t\right){p}^{\mathrm{others}}\left(t\right)\right]\frac{I\left(t\right)}{N\left(t\right)},$$

which sets a different contact rate and secondary attack rate at home, school, workplace, and others respectively. Economic models assume some situations are related to economic activity. In earlier simulation studies, Eichenbaum et al. (2020, 2021) and Jones et al. (2020) employed

26 $$\begin{array}{c}{\text{New}}\left(t\right)=\beta \left(t\right)S\left(t\right)I\left(t\right)={\beta }_{0}\left[\left(1-\alpha \right)+\alpha {\left(1-y\left(t\right)\right)}^{2}\right]S\left(t\right)I\left(t\right),\end{array}$$

where the transmission rate is the quadratic function of economic activity. The simple SIR model can be considered as the case of $\alpha =0$.

Eichenbaum et al. (2020, 2021) set the transmission opportunities to be 1/3 each in the home, community, and school/workplace, with half of the community contacts related to consumption and half of the school/workplace contacts related to labor supply. They assume that contact in the home does not increase or decrease, consumption and labor supply are proportional, and maximum suppression of economic activity (which is unrealistic because of the total cessation of economic activity) can reduce contact opportunities by up to 1/3. When the economic activity is restrained by *y*, the transmission rate becomes

$$\beta \left(t\right)={\beta }_{0}\left[\frac{2}{3}+\frac{1}{3}{\left(1-y\left(t\right)\right)}^{2}\right].$$

The suppression works in a squared manner because each side of contacting persons restrain their activities. This can also be interpreted as assuming density-dependent contact.

Meanwhile, Jones et al. (2020) assumed that suppression of consumption can reduce up to all local contacts and suppression of labor supply can reduce up to all contacts in schools and workplaces. They assumed that restricting economic activity would not change household contact, so they assumed that restricting economic activity would reduce contact opportunities by up to two-thirds. In this case, the transmission rate becomes

$$\beta \left(t\right)={\beta }_{0}\left[\frac{1}{3}+\frac{2}{3}{\left(1-y\left(t\right)\right)}^{2}\right].$$

### Dynamic externalities

Next, consider the implications of ${\lambda }_{I}$. The dynamics of co-state variables is

27 $$\begin{array}{c}\dot{{\lambda }_{I}}\left(t\right)-\left(\rho +\nu \right){\lambda }_{I}\left(t\right)=-\frac{\partial H\left(t\right)}{\partial I\left(t\right)}=-\phi \left(I\left(t\right)\right)\gamma \text{VSL}-{\phi }^{\prime}\left(I\left(t\right)\right)\gamma I\left(t\right)\text{VSL}-{\lambda }_{I}\left(t\right)\frac{\partial \mathrm{New}\left(t\right)}{\partial I\left(t\right)}\\ +{\lambda }_{I}\left(t\right)\gamma -{\lambda }_{R}\left(t\right)\gamma +{\lambda }_{N}\left(t\right)\phi \left(I\left(t\right)\right)\gamma +{\lambda }_{N}{\phi }^{\prime}\left(I\left(t\right)\right)\gamma I\left(t\right),\end{array}$$

28 $$\begin{array}{c}\dot{{\lambda }_{R}}\left(t\right)-\left(\rho +\nu \right){\lambda }_{R}\left(t\right)=-\frac{\partial H\left(t\right)}{\partial R\left(t\right)}=-{\lambda }_{I}\left(t\right)\frac{\partial {\text{New}}\left(t\right)}{\partial R\left(t\right)},\end{array}$$

29 $$\begin{array}{c}\dot{{\lambda }_{N}}\left(t\right)-\left(\rho +\nu \right){\lambda }_{N}\left(t\right)=-\frac{\partial H\left(t\right)}{\partial N\left(t\right)}=-y\left(t\right)\overline{Y }.\end{array}$$

Solving Eqs. (27), (28), and (29) forward yields

30 $${\lambda }_{I}\left(t\right)={\int }_{t}^{\infty }{\mathrm{e}}^{-\left(\rho +\nu +\gamma \right)\left(s-t\right)}\left[\left(\mathrm{VSL}-{\lambda }_{N}\left(s\right)\right)\phi \left(I\left(t\right)\right)\gamma +\left(\mathrm{VSL}-{\lambda }_{N}\left(s\right)\right){\phi }^{\prime}\left(I\left(t\right)\right)\gamma I\left(t\right)+{\lambda }_{I}\left(s\right)\frac{\partial \mathrm{New}\left(s\right)}{\partial I\left(s\right)}+{\lambda }_{R}\left(s\right)\gamma \right]\mathrm{d}s$$

$${\lambda }_{R}\left(t\right)={\int}_{t}^{\infty }{\mathrm{e}}^{-\left(\rho +\nu \right)\left(s-t\right)}{\lambda}_{I}\left(s\right)\frac{\partial \mathrm{New}\left(s\right)}{\partial R\left(s\right)}\mathrm{d}s$$

$${\lambda }_{N}\left(t\right)={\int }_{t}^{\infty }{\mathrm{e}}^{-\left(\rho +\nu \right)\left(s-t\right)}y\left(s\right)\bar{Y}\mathrm{d}s$$

Note that Eq. (30) has not yet been solved for ${\lambda }_{I}$, because ${\lambda }_{I}$ remains at the right-hand side of (30). Since the individuals do not think their behavior affects the state variables, the effect of *I* on (30) creates the dynamic externality, as extensively discussed by Garibaldi et al. (2020b) and Gonzalez-Eiras and Niepelt (2020b).^15^ The terms in the bracket on the right-hand side of (30) have the following implications. The first term is the expected value of fatality loss of a newly infected person. It is not a source of externality, because private agents recognize it. In contrast, the last three terms consist of dynamic externalities. The second term (medical congestion externality^16^) represents the costs of additional deaths under the strong stress on the medical capacity. The third term (contagion externality) reflects that the new infection will increase the future infection by increasing the infectious people. The fourth term (immunity externality) reflects that the current new infection will decrease the future infection after newly infected people get immunized.

The impacts of these externalities vary over time. The medical congestion externality, which is always negative, becomes high at the peak of epidemic (high *I*); when the medical capacity is tight, additional patients raise the fatality rate, and agents do not recognize this effect in choosing their prevention measures.

The contagion externality is negative because it works to accelerate the spread of disease. The evolution of this externality is intuitively captured by looking at the simple case of constant transmission rate ($\alpha =0$, in (26)). The contagion externality is driven by $\partial \mathrm{New}/\partial I={\beta }_{0}\left(S-I\right)$. The effect of increasing infection is strong when there are many susceptible people; an infectious person is more likely to contact a susceptible person. Since *S* gradually declines, the negative contagion externality is likely to be large at an early stage of the epidemic and becomes small later. Of course, the accurate path of externality should be grasped in the case of time-varying transmission rate.

However, the immunity externality is positive. After newly infected people get immunized, they contribute to suppressing the spread of infection and achieving a herd immunity. Under a constant transmission case, this externality is driven by $\partial \mathrm{New}/\partial R=-{\beta }_{0}I.$ This effect is strong when the infection is prevailing.

The overall effects of externalities can be assured only by numerical simulations because these externalities are time-varying and offsetting. Many studies (e.g., Garibaldi et al., 2020b; Gonzalez-Eiras & Niepelt, 2020b; Kubota, 2021; Makris & Toxvaerd, 2020; Phelan & Toda, 2021) found that the overall externalities including static externality can be positive in a later stage of the epidemic. The positive externality creates a motive to introduce an “inverse lockdown” to promote the infection, because the social cost of infection is smaller than the private cost. When the herd immunity becomes foreseeable, susceptible people hope they will see the end of the epidemic and be uninfected; their behavior becomes too cautious from the society’s viewpoint. The predictable timing of vaccines becomes another important source of dynamic externalities, although this paper ignores it by assuming constant conditional probability of vaccine availability.

### Timing of lockdown

Using Eq. (25), let us consider the timing of the lockdown. We can think of the lockdown as the peak time of the suppression of economic activity. First, the marginal cost of lockdown is smaller with a smaller population; therefore, the more it is delayed, the lower the marginal cost. Since the impact of deaths on the total population is not very large, however, this evolution of marginal cost is not crucial to determine the timing. Meanwhile, the marginal benefit is large when the new infection is high ($S\left(t\right)I\left(t\right)$ is large). This is because from (26),

$$\frac{\partial \mathrm{New}\left(t\right)}{\partial \left(1-y\left(t\right)\right)}=2{\beta }_{0}\alpha S\left(t\right)I\left(t\right)\left(1-y\left(t\right)\right).$$

In the SIR model, suppressing infectious people and increasing the number of the removed will reduce the long-term number of infected people and deaths. Since lockdowns have the effect of reducing the rate of infection by a certain percentage, this long-term effect will be greater during periods of high prevalence rate (the ratio of infectious to total population). Therefore, implementing a costly lockdown is appropriate when the number of infectious people has increased to some extent.

However, the existence of vaccines creates a benefit of early implementation, making it desirable to implement the lockdown before the peak of the epidemic. In the objective function, the benefit of early action is increased by adding the probability *ν* that a vaccine will be developed at each point in time and discounting the future.

Whether or not the lockdown has actually been effective is a separate issue. Verifying the effects is difficult because of several reasons. Since countermeasures are endogenously determined, countries with epidemics are more likely to adopt lockdowns. However, this does not mean that lockdowns are causally related to the epidemic’s severity. The effects of lockdowns are difficult to isolate when less costly voluntary restraint and milder social distancing are taken before lockdowns.

### Other model specifications

Since the optimal infectious disease control dynamics in the SIR model cannot be solved explicitly, the analysis must rely on numerical simulations. In this paper, instead of using the standard SIR model as it is, we use an approximate model that excludes changes in the population from the new infection function (20) to make it easier to track the effects of infectious disease control. Along these lines, Gonzalez-Eiras and Niepelt (2020a) analytically find policy solutions by using the tractable model that approximates the SIR model.

The SIR model assumes immunity is permanent, but adoptive immunity to the virus that causes COVID-19 is not permanent and reinfection occurs. Therefore, perhaps the susceptible-infected-susceptible (SIS) model should be used, where the patient reverts to a susceptible person who may be infected again after infection. Giannitsarou et al. (2020) have shown the importance of taking measures from the beginning in the susceptible-exposed-infected-susceptible (SEIRS) model^17^ because infected people do not acquire permanent immunity. The disease will become endemic in the long term, with a certain persisting number of new infections.

### Ex-ante assessment: simulation analysis of lockdown

Early studies on the lockdown had conducted in Spring 2020, when many countries have introduced lockdown, stay-at-home order, or social distancing. Since the setting of many parameters cannot be based on experiences, they belong to ex ante assessment. We briefly review Alvarez et al. (2020), Eichenbaum et al. (2021), Krueger et al. (2020) and Jones et al. (2020). Those studies focus on a mitigation policy, which tries to slow the spread of infectious disease and suppress the peak of infection.

Eichenbaum et al. (2021) set the basic reproduction number 1.5, which can be reduced to 1 by shutting down all economy activities. Since shutting the whole economy down brings prohibitive costs, a rational solution is mitigation, which allows the basic reproduction number to be above 1. They contrast when a representative individual takes action to prevent infection (basic SIR-macro model) and when no action is taken (SIR model). When vaccines are not considered, prevention behavior can reduce the peak of the infection, but the drop in consumption can be up to 10%.

They also compare the case of private actions alone (Benchmark SIR-macro model) and the case of optimizing society as a whole (Best Simple Containment Policy), where externalities of infection and externalities of healthcare resource constraints exist. The difference between the two cases represents the effect of NPI, which amounts the reduction of consumption by 30%. Their simulation also suggests that prevention measures should be implemented more aggressively from the beginning, when the model takes account of development of vaccine. In addition to vaccines, the simulation takes in account the development of treatments.

Alvarez et al. (2020) set the basic reproduction number 3.6. Lockdown can lower it by 57.75%. Their optimal plan is to reduce economic activity sharply after about three weeks from the onset of epidemic. They also conduct a sensitivity analysis regarding the value of a statistical life. Although the figures are obtained from the dynamic optimization of the SIR model with a system of non-linear differential equations, they show an almost proportional relationship between the value of a statistical life and the economic loss due to lockdown. In addition, the reduced human loss due to lockdown is estimated to be almost double the economic loss.

Jones et al. (2020) sets the basic reproduction number at 2, which can be reduced to 0.67 (67% reduction) if all economic activities are shut down. We can keep the effective reproduction number less than 1, while avoiding a full shutdown. However, the optimal policy lets it above 1, thus observing a gradual spread of the epidemic, although the epidemic curve is flattened. Their simulation show a very large suppression of infectious people, compared to Eichenbaum et al. (2021). Consumption drops by more than 30%. They also take account of work from home to prevent infection and find that if work from home is possible, almost 80% of labor force work from home and the drop of consumption becomes almost 45%. Although the model details differ, it can be considered a sensitivity analysis on the efficiency of lockdown compared to the analysis of Eichenbaum et al. (2021).

Although consumption at the prevailing epidemic is substituted across time, it can be substituted within time in the real world (e.g., from shopping at a store to shopping online, or from a contact sport to an e-sport), thus preventing the infection with less drop of consumption. Krueger et al. (2020) modeled many consumption goods that differ in contagiousness. Their simulation found that the drop of consumption is significantly smaller than in a single consumption good model of Eichenbaum et al. (2021), because the intra-temporal reallocation of consumption can reduce the risk of infection.

## Ex post evaluation of lockdown

### Cost–benefit analysis

Since the latter half of 2020, studies that analyze the costs and benefits of actual countermeasures against COVID-19 have emerged. Broughel and Kotrous (2021), Doti (2021), Scherbina (2021) and Thunstrom et al. (2020) study stay-at-home orders of the USA, and Miles et al. (2020, 2021) and Rowthorn and Maciejowski (2020) focus on the lockdown in the UK.^18^ In addition, based on the experiences in 2020, Scherbina (2021) evaluates the costs and benefits of introducing lockdown in early 2021.

Ex post cost–benefit analysis calculates the net social benefit (NSB), which is the difference in the monetized social welfare between the economy under the actual policy and the “no action is taken” counterfactual. Their results on the sign of NSB are not conclusive; however, this is not due to the sensitivity analysis of a single study not providing a robust conclusion. The signs are supported by some studies that have obtained a robust result with sensitivity analysis. Therefore, the contents of each study must be examined in depth. We review the estimates of costs, effects and benefits. Although the presentation of estimates varies widely among studies, we reorganize them as comparable as possible. To this end, we compare their estimates of incremental cost–effectiveness ratio (ICER), which is the ratio of the incremental effect (consequences of the reduction in new infections) to the incremental cost of strengthening a countermeasure. If the analysis takes account of only lives saved as an effect of policy, the benefit becomes the product of lives saved and the VSL.

The ICER and VSL have analogs in the model expressed in Sect. 5. When an infinitesimal policy change at time *t* is considered, the ratio of the marginal effect of reducing new infections to the costs of doing it is an analog of ICER, which can be represented as

$$\mathrm{ICER}\left(t\right)\equiv N\left(t\right)\overline{Y }/\frac{\partial \mathrm{New}\left(t\right)}{\partial \left(1-y\left(t\right)\right)}.$$

Multiplying the reciprocal of this “ICER” by the social cost of new infectious, ${\lambda }_{I}$, yields the analogue of benefit–cost ratio, B/C, which is used by the cost–benefit analysis. Thus, the analogs of ICER and B/C are connected with

$$\frac{B\left(t\right)}{C\left(t\right)}\equiv \frac{{\lambda }_{I}\left(t\right)\frac{\partial \mathrm{New}\left(t\right)}{\partial \left(1-y\left(t\right)\right)}}{N\left(t\right)\overline{Y} }=\frac{{\lambda }_{I}\left(t\right)}{\mathrm{ICER}\left(t\right)},$$

where *B* is the benefit and *C* is the cost. The optimal policy satisfies $B\left(t\right)/C\left(t\right)=1$, as in Eq. (25).

Practical calculations differ from the analogs of the model. First, actual estimates aggregate the impacts of the measures over a certain period. Second, since ${\lambda }_{I}$ depends on the entire path of the dynamic model as shown in (30), it applies hardly to ex post policy evaluation. Existing studies approximate it as the VSL of deaths. Then the ratio of VSL to ICER is the benefit–cost ratio; if VSL is greater than ICER, the benefit–cost ratio is greater than 1, and the countermeasure is valuable. Conversely, if the ICER is greater than the VSL, the benefit–cost ratio is less than 1 and the countermeasure is not justified.

### Back-of-the-envelope calculation

Let us start from Miles et al. (2020, 2021). They used a relatively simple method to calculate the costs and benefits of lockdown in the UK, and showed that its costs exceed the benefits over various scenarios.

The costs are identified as a reduction in GDP, and are set at 9–25% of GDP based on the UK’s experience and forecast. If the economic downturn seen after the epidemic is a result of government and private sector actions to avoid infection, this approach would obtain a reasonable estimate.

The effect is considered to be a reduction in deaths, and is determined by the difference between the actual experiences and the estimate of 500,000 deaths under the “no action is taken” scenario by the Imperial College team (Ferguson et al. 2020). As long as we admit the structure of the SIR model, the estimate of the death if no action is taken is determined by the basic reproduction number and the fatality rate, and the accumulation of research has shaped a reliable range. However, this estimate is regarded as an upper limit. If the heterogeneity of individuals is considered, the basic reproduction number will not remain constant but will decline, and the number of deaths will decrease.

Miles et al. (2020, 2021) set the lower bound of the effect (reduction in deaths) at 20,000 and the upper bound at 440,000 (500,000 minus 60,000 actual deaths) using the method described above. They arbitrarily set the lower bound without a solid evidence. The lower bound of the ICER (the combination of the lower bound of the cost estimate and the upper bound of the effectiveness estimate) is calculated to be about £454,000. Assuming that the countermeasure has an average life-prolonging effect of 10 years, and assuming a VSL of £300,000 for 10 living years used by the UK National Institute for Health and Clinical Excellence (NICE), the ICER exceeds the VSL and the lockdown is not justified.

The information needed in this estimation is minimal: actual and projected GDP, projected death of SIR model, and realized deaths. Although the procedure is simple, the root of the problem can be easily traced, if we obtain an invalid estimate. If the policy is justified (ICER is less than VSL) by other “sophisticated” methods, one of the following may have occurred in comparison with the back-of-the-envelope calculation:

1. the cost of the measure is smaller than the actual decline in GDP (other factors contribute to the decline in GDP),
2. the effect is greater, or
3. the value of a prolonged life is greater.

As will be discussed, other studies tend to justify the countermeasures. Let us examine the reasons why.

### Estimates of costs

First, we look at the cost estimates. Rowthorn and Maciejowski (2020) and Doti (2021) formulated the relationship between costs and effects, whereas other studies estimated the effects and costs independently. Doti (2021) uses cross-sectional data from US states to estimate the effects and costs of stay-at-home orders in a setting where the relationship between the two is linked. The SIR model of Rowthorn and Maciejowski (2020) specifies the relationship between effects (reduction of transmission rate) and costs as

$$\beta \left(t\right)={\beta }_{0}\left[1-1.064{y\left(t\right)}^{1/3}\right].$$

This implies that the maximum restraint is 35% of GDP, and with this maximum reduction, the transmission rate will decline by 75% (the effective reproduction number will drop from 3 to 0.75).^19^ The costs of countermeasures are calculated inside the model.

**Fig. 1:**
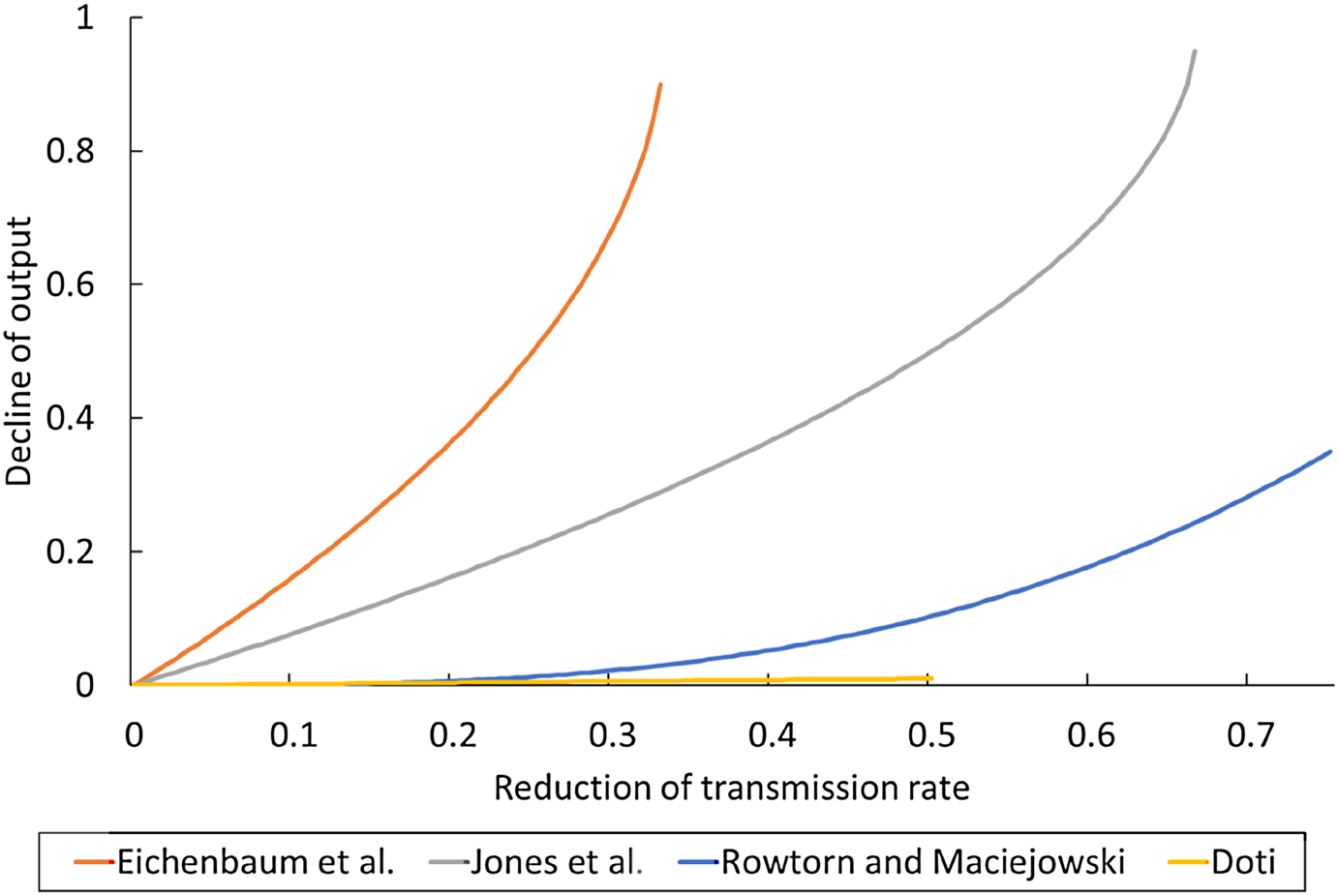
Settings of reduction of transmission occasion and its costs. The horizontal axis is the reduction rate of transmission rate. The vertical axis is the reduction rate of output

Figure 1 shows the decrease in transmission rate at the horizontal axis and the decrease in GDP at the vertical axis. Their setting derives lower costs than the settings of earlier simulations like Eichenbaum et al. (2021) and Jones et al. (2020).^20^

Broughel and Kotrous (2021) and Scherbina (2021) review existing estimates of costs of stay-at-home orders in the USA, respectively. Then, they set similar figures for the cost per week; $357 billion by Broughel and Kotrous (2021) and $369.3 billion by Scherbina (2021). Thunstrom et al. (2020) included the future decline in GDP in their costs, resulting in an estimate of $7.21 trillion, a much larger figure than the other studies.

### Estimates of effects and benefits

Scherbina (2021), Rowthorn and Maciejowski (2020), and Thunstrom et al. (2020) estimate the effect of countermeasures by using the SIR model’s predictions of deaths with lockdown, rather than the experiences. This method estimates the number of infected people that will decrease as the infection rate decreases during the period of lockdown. As the duration of the lockdown increases, the reduction in the number of infected people diminishes. Assuming that the marginal cost of extending the period of the lockdown is constant, the optimal duration of the lockdown can be determined at the point where the marginal benefit of reducing new infection equals its marginal cost.

Broughel and Kotrous (2021) and Scherbina (2021) considered the loss of quality of life due to morbidity and long COVID as well as the reduction in mortality losses. We should note that focusing only on fatality losses will result in underestimation because the latter occupies a nonnegligible part of total benefit. According to Broughel and Kotrous (2021), mortality reduction benefits are estimated to be $317.7–$331.5 billion, whereas the total benefits are estimated to be $632.5–$765 billion. Broughel and Kotrous' (2021) estimate of the reduction in mortality losses belongs to the back-of-the-envelope calculation described above.

### Estimates of incremental cost–effectiveness ratio and setting of value of a statistical life

Table 2 summarizes the ICER, VSL settings, and NSB estimates of the studies described earlier.^21^ The ICER of Thunstrom et al. (2020), which had the largest cost estimate, is notably large, but at the same time their setting of VSL is large, resulting in the largest net benefit estimate. Broughel and Kotrous (2021) and Scherbina (2021) estimated a positive net benefit, too.

**Table 2:** Comparison of incremental cost–effectiveness ratio and value of a statistical life

	Cost per capita	Effect per 1000 persons	ICER	VSL	NSB (billion)
(United States)					
Broughel and Kotrous	*649*	2.85	*228,000*	338,000	301.0–550.8
Doti	*1241*	1.08	1,145,000	4,200,000	(N.A.)
Scherbina	*447*	*0.62*	*726,000*	*5,639,000*	653.1
Thunstrom et al.	*21,831*	3.75	*5,819,000*	10,000,000	5160
(United Kingdom)					
Miles et al.	*2986*	6.58	*454,000*	300,000	− 547 to − 68
Rowthorn and Maciejowski	*1060*	5.68	*187,000*	2,000,000	(N.A.)

The unit is US dollars and UK pounds. Italic indicates the value not reported in the reference, but complemented by the author. “Effect” indicates the lives saved by countermeasures against COVID-19

**Fig. 2:**
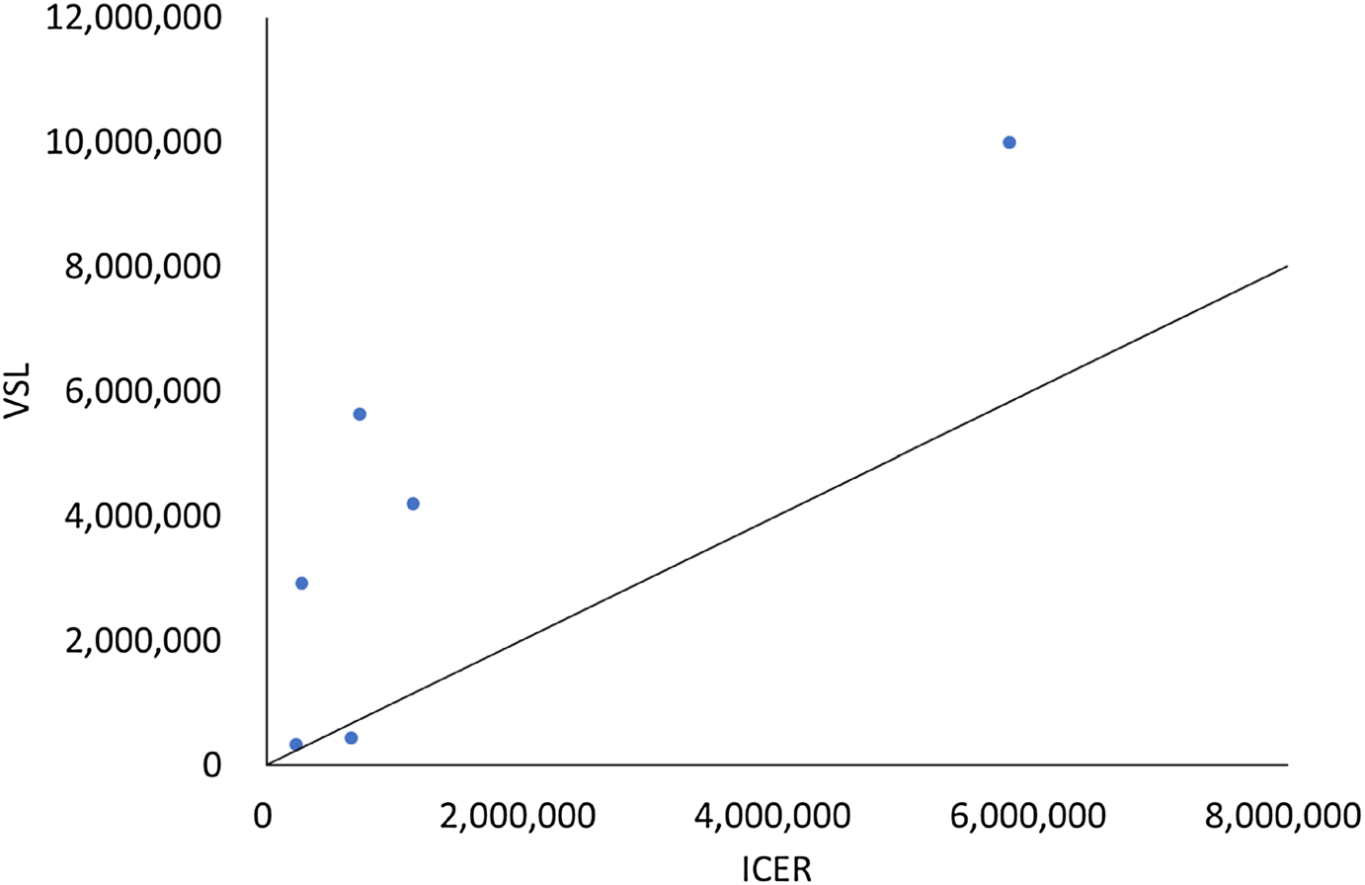
Incremental cost–effectiveness ratio and value of a statistical life. Unit of both axes is US dollar. The horizontal axis is ICER. The vertical axis is VSL. On the straight line, ICER and VSL are equal. On the upper left of the straight line, VSL is greater than ICER and B/C is greater than 1. At the bottom right of the line, ICER is greater than VSL and B/C is less than 1

Figure 2 shows the relationship between the two, with ICER on the horizontal axis and VSL on the vertical axis. The estimates for the UK are converted to US dollars by using the OECD’s estimate of purchasing power parity in 2019. The range of VSL settings is much wider than the range of ICER estimates in all studies, except that of Thunstrom et al. (2020), because of the following: First, a wide range of VSL settings are cited. Although VSLs set by public institutions are often cited, as Robinson (2007) highlighted, a large range exists in the values set for VSLs by public institutions. Therefore, citing the values set in public institutions may result in a large range of VSL. Second, as shown in Sect. 4.3, the existing estimate of VSL is derived from the attitudes toward a very small risk, and may overestimate an appropriate measure in the case of COVID-19. Such a consideration is missed by some studies. Thirdly, a difference exists in the assumption of extended life expectancy: the life expectancy assumed by VSL is the life expectancy of the total population, but the life expectancy of those who die of COVID-19, which is biased toward the elderly, is shorter than this. Fourthly, if lockdown measures are taken again later (indeed, they were taken), the effect of saving lives by the earlier lockdown will only go so far. Therefore, the direct usage of life expectancy leads to a significant overestimation. Except for the first reason, the wide range of VSL may contain a large, upward bias.

### What we got to know

Studies that have estimated the costs and benefits of infectious disease control measures implemented in 2020 have reached mixed conclusions as to whether the measures are warranted or not. Since the estimation of costs can utilize actual observations, the estimate is relatively stable. If the effect is calculated from the SIR model, we can expect a relatively stable result, too. However, assuming uniform contacts among homogeneous individuals may lead to overestimation. In contrast, the setting of VSL for converting effects into benefits has a very wide range, and are also likely to lead to overestimation of benefits. Therefore, a careful procedure for setting VSL is crucial for obtaining a reliable evaluation of countermeasures against COVID-19.

The studies look only at the aggregates in evaluating economic losses, thus ignoring the welfare losses related to inadequate allocations of consumption among individuals and among types of consumption. Evaluating these welfare losses will be an important task for future studies.^22^

## Testing and isolation

### Uniform testing

Although the lockdown uniformly restricts the activities of everyone, this section and the following one examines the efficiency of measures that limit the activities of only a small part of population. Testing and isolation, which is examined in this section, attempts to restrict the activities of only the infectious. Since the proportion of infectious people in the population is small, the restrictions required to achieve the same effect of preventing the spread of infection are expected to be far less than in a lockdown.

First, we consider measures to test a portion *τ* of the population and isolate those who test positive (uniform testing). We then focus on contact tracing, which effectively isolates positive individuals by tracing the contact history of the infected and identifying those who had a close contact with the infected.

For simplicity, we assume no error in testing.^23^ Assume that the proportion *ι* of infectious is isolated, and is no longer a source of new infection. The isolated infectious, $\tilde{I }$, is defined by

$$\tilde{I }\left(t\right)\equiv \iota \left(t\right)I\left(t\right).$$

The dynamics of infectious is slightly changed from (5) to

31 $$\begin{array}{c}\dot{I}\left(t\right)={\text{New}}\left(1-y\left(t\right),\left(1-\tau \left(t\right)\right)\left(I\left(t\right)-\tilde{I }\left(t\right)\right),R\left(t\right)\right)-\gamma I\left(t\right).\end{array}$$

Here the new infectious is infected from those not isolated after tested. The dynamics of isolated infectious is given by

32 $$\begin{array}{c}\dot{\tilde{I }}\left(t\right)=\tau \left(t\right)\left(1-\gamma \right)\left(I\left(t\right)-\tilde{I }\left(t\right)\right)-\gamma \tilde{I }\left(t\right).\end{array}$$

For the sake of simplicity, marginal cost of test is constant and denoted by *c*. This cost contains not only monetary costs, but also examinee’s opportunity cost of time and psychic costs. We assume that the disutility of isolated persons and output loss associated with isolation are small and negligible, and that *c* is considered as only economic costs. Monetized welfare loss is then

33 $$\begin{array}{c}\frac{{V}_{0}-V\left(0\right)}{{u}^{{{\prime}}}\left(\overline{Y }\right)}={\int }_{0}^{\infty }{\mathrm{e}}^{-\left(\rho +\nu \right)t}\left[N\left(t\right)y\left(t\right)\overline{Y }+c\tau \left(t\right)\left(N\left(t\right)-\tilde{I }\left(t\right)\right)+\phi \left(I\left(t\right)\right)\gamma I\left(t\right)\mathrm{VSL}\right]\mathrm{d}t.\end{array}$$

Since the loss of production due to the infected individual’s inability to work is ignored in Eq. (11), the loss of production due to the inability of the quarantined to work is also discarded. In addition, we only consider the tests for isolation and discard the tests required for medical treatment. In the model, testing may not be necessary because the recovering person will gain immunity, but he or she may be subject to testing in the real world. Equation (33) assumes that they will be subject to testing.^24^

With the above setup, a dynamic optimization problem similar to the lockdown can be formulated as a problem of minimizing the monetized welfare loss, Eq. (33), with the dynamics of the state variables, Eqs. (22), (23), (31), and (32), as constraints, and *y* and *τ* as policy variables. The study of Chari et al. (2020), presented below, belongs to such a setting. Since testing facilities and technicians are practically fixed production factors, assuming that the number of tests can be freely changed at each point in time is unrealistic. Therefore, a different setting that describes measures that can actually be implemented must be considered.

The expansion of testing can ease the restrictions on economic activities while preventing infection, thus mitigating the trade-off between health and economy analyzed in Sect. 5. However, as for the testing itself, there is a trade-off between health and economy, as the expansion of the testing will prevent infection, but the cost will be a burden on the economy.

### Contact tracing

Contact tracing is a measure to prevent the spread of infection by tracing the contact history of infectious persons and detecting possible secondary cases to prevent further infections. Since the positivity rate of the test of close contacts is much higher than that of uniform testing, it is much more efficient in detecting those who test positive. Since contact tracing tests only a subset of the population, however, it misses other infectious persons who are not its target.

The efficiencies of uniform testing and contact tracing can be compared as follows. The rate of positivity of uniform testing, which can be considered as random sampling, is

34 $$\begin{array}{c}\frac{\tau \left(1-\iota \right)I}{\tau \left(1-\iota \right)I+\tau S}=\frac{\left(1-\iota \right)I}{\left(1-\iota \right)I+S}.\end{array}$$

If contact tracing captures the positive person by the factor of *θ* compared with random sampling, the ratio of infectious to susceptible in the tested group becomes $\theta I/S$ instead of $I/S$ of uniform testing, and the rate of positivity becomes

35 $$\begin{array}{c}\frac{\left(1-\iota \right)\theta I}{\left(1-\iota \right)\theta I+S}=\frac{\left(1-\iota \right)I}{\left(1-\iota \right)I+{\theta }^{-1}S}.\end{array}$$

From (34) and (35), the relative ratio of positive tests between contact tracing and uniform testing is

$$\frac{\left(1-\iota \right)I+S}{\left(1-\iota \right)I+{\theta }^{-1}S}=\frac{1+\left(1-\iota \right)I/S}{{\theta }^{-1}+\left(1-\iota \right)I/S}.$$

If the positivity rate is sufficiently small, the positivity rate for contact tracing is almost *θ* times larger. Since the reciprocal of the positivity rate is the number of people tested per positive person, the cost of testing can be reduced to almost the reciprocal of *θ* in contact tracing than in uniform testing. However, in contact tracing, the cost savings are smaller than the inverse of *θ* because the cost of increasing *τ* is not only the cost of testing, but also the cost of tracking down close contacts.

Implementing uniform testing and contact tracing simultaneously is practical rather than choosing either. In this case, shifting resources for testing from contact tracing to uniform testing is inefficient because the probability of catching positive individuals is much higher in contact tracing.

The positivity rate of uniform testing is considerably smaller than when contact tracing is conducted. Therefore, in such a situation, the degree to which uniform testing is necessary can be determined as follows: (1) not necessary (marginal benefits are already below marginal costs at the stage of introducing uniform testing), (2) necessary at a certain scale (marginal benefits exceed marginal costs at the stage of introducing uniform testing, but at the stage of expanding uniform testing, marginal benefits and marginal costs become equal),^25^ or (3) testing everyone is necessary (marginal costs exceed marginal benefits until everyone is tested). Which of these cases holds seems to be yet to be determined so far.

### Simulation analysis of uniform testing and contact tracing

Chari et al. (2020) compared no testing, uniform testing, and contact tracing under the assumption that the testing capacity at each time point can be set freely. Based on data from a positive epidemiological survey in Korea, they set *θ* at 86. The constant decline in consumption is used as an indicator of welfare loss. For the uniform inspection, the welfare improvement is only 0.12% of consumption, which is negligible compared to no inspection, but for the contact tracing, the improvement is 2.48% of consumption. They show that the contact tracing can keep the reproduction number $\beta \left(t\right)/\gamma$ low, and the suppression of economic activities is also kept low.

The optimal number of tests fluctuates significantly, but it would be difficult to implement such flexible testing as mentioned above. This finding is another disadvantage of relying on uniform testing.

The number of tests in contact tracing fluctuates more smoothly. Therefore, the hurdle to maintaining the testing capacity to conduct this is lower than that of uniform testing. Note also that contact tracing capacity may limit it, and if the number of infectious people exceeds the capacity to be tracked, the contact tracing will not be able to be effective.

Chari et al. (2020) also analyzed quarantine measures without testing,^26^ and they estimated a welfare improvement equivalent to 1.41% of consumption compared to uniform testing. This result allows us to distinguish between the effects of testing and quarantine. The purpose of testing is to eliminate the error of quarantining those who test negative, and the welfare improvement from this is substantial, but the welfare improvement from quarantining those with a high probability of infection is also significant.

In the early stages of an epidemic, the lack of a testing capacity (and the prohibitively high cost of expanding testing in the short term) forces the use of uniform activity restrictions, which are less efficient, but given time to develop a testing capacity, it may be more efficient to switch to testing and isolation. Eichenbaum et al. (2020), who provided analysis in this context, conduct a simulation analysis comparing private prevention and uniform testing in a setting where testing capacity is not freely set but gradually expanded. The time unit in this model is week, and the testing capacity is assumed to expand each week so that *α* of the population enters the population to be tested each week. Two types of testing and isolation are considered: one in which positive individuals’ workplace and consumption contacts (one-third of the total) are restricted (smart containment), and one in which all contacts are restricted (strict containment).

Eichenbaum et al. (2020) compared market equilibrium (voluntary prevention behavior by the private sector) and uniform testing, and shows that uniform testing greatly reduces the decline in consumption. From the comparison with the same authors’ analysis of lockdown (Eichenbaum et al., 2021), the drop in consumption due to uniform testing is much smaller than that of lockdown.

A superiority of testing and isolation over lockdown is also found in Piguillem and Shi (2020), which calibrated the Italian outbreak. Uniform testing (targeted isolation) and lockdown (indiscriminate quarantine) can be conducted simultaneously, although their simulation restricts the extent of lockdown and test to a linear, increasing function of the prevalence rate. When uniform testing is combined, the optimal extent of lockdown substantially shrinks, because the testing and isolation strategy removes infectious people with substantially smaller costs. They observed that uniform testing is a close substitute for lockdown.

## Multidimensional SIR model

### Basic reproduction number in multidimensional SIR model

The SIR model, which assumes that all individuals are homogeneous, cannot describe the phenomenon that the morbidity and mortality of COVID-19 vary greatly with age. A multidimensional SIR model, in which individuals are classified into different social groups, can depict such an important phenomenon. This section presents a model in which the individuals in the model are classified into *n* age groups. Let us denote the age group with the subscript *i*. Individuals in one age group are infected by contact with individuals in each age group. New infectious of age group *i* is

36 $$\begin{array}{c}{\mathrm{New}}_{i}\left(t\right)={S}_{i}\left(t\right){C}_{i1}\left(t\right){p}_{i1}\left(t\right)\frac{{I}_{1}\left(t\right)}{{N}_{1}\left(t\right)}+\dots +{S}_{i}\left(t\right){C}_{in}\left(t\right){p}_{in}\left(t\right)\frac{{I}_{n}\left(t\right)}{{N}_{n}\left(t\right)}.\end{array}$$

The dynamics of infectious is

37 $$\begin{array}{c}{\dot{I}}_{i}\left(t\right)={\mathrm{New}}_{i}\left(t\right)-\gamma {I}_{i}\left(t\right).\end{array}$$

The number of contacts that a person in age group *i* makes with persons in age group *j* is denoted by ${C}_{ij}$. The ratio of infectious to contacts (secondary attack rate from age group *j* to age group *i*) is denoted by ${p}_{ij}$. Substituting (36) into (37) and using vectors and a matrix yields

38 $$\begin{array}{c}\left[\begin{array}{c}{\dot{I}}_{1}\left(t\right)\\ \vdots \\ {\dot{I}}_{n}\left(t\right)\end{array}\right]=\left[\begin{array}{ccc}{S}_{1}\left(t\right){C}_{11}\left(t\right){p}_{11}\left(t\right)/{N}_{1}\left(t\right)& \dots & {S}_{1}\left(t\right){C}_{1n}\left(t\right){p}_{1n}\left(t\right)/{N}_{n}\left(t\right)\\ \vdots & \ddots & \vdots \\ {S}_{n}\left(t\right){C}_{n1}\left(t\right){p}_{n1}\left(t\right)/{N}_{1}\left(t\right)& \dots & {S}_{n}\left(t\right){C}_{nn}\left(t\right){p}_{nn}\left(t\right)/{N}_{n}\left(t\right)\end{array}\right]\left[\begin{array}{c}{I}_{1}\left(t\right)\\ \vdots \\ {I}_{n}\left(t\right)\end{array}\right]-\gamma \left[\begin{array}{c}{I}_{1}\left(t\right)\\ \vdots \\ {I}_{n}\left(t\right)\end{array}\right].\end{array}$$

Epidemiological studies use the “next-generation matrix” and define the basic reproduction number by the next-generation matrix (Diekmann et al., 1990; van den Driessche & Watmough, 2002). However, the present paper harmonizes with the framework in economics and does not use the next-generation matrix, and sticks to the SIR model.

Linear approximation of (38) at the pre-epidemic state (no infectious, all susceptible) becomes

39 $$\begin{array}{c}\left[\begin{array}{c}{\dot{I}}_{1}\left(t\right)\\ \vdots \\ {\dot{I}}_{n}\left(t\right)\end{array}\right]=\left[\begin{array}{ccc}{C}_{11}{p}_{11}{N}_{1}\left(0\right)/{N}_{1}\left(0\right)& \dots & {C}_{1n}{p}_{1n}{N}_{1}\left(0\right)/{N}_{n}\left(0\right)\\ \vdots & \ddots & \vdots \\ {C}_{n1}{p}_{n1}{N}_{n}\left(0\right)/{N}_{1}\left(0\right)& \dots & {C}_{nn}{p}_{nn}{N}_{n}\left(0\right)/{N}_{n}\left(0\right)\end{array}\right]\left[\begin{array}{c}{I}_{1}\left(t\right)\\ \vdots \\ {I}_{n}\left(t\right)\end{array}\right]-\gamma \left[\begin{array}{c}{I}_{1}\left(t\right)\\ \vdots \\ {I}_{n}\left(t\right)\end{array}\right],\end{array}$$

where ${C}_{ij}$ and ${p}_{ij}$ are a constant evaluated at the pre-epidemic state and the susceptible becomes the population. Transforming (39) by using matrices and vectors and letting *I* an identity matrix yields

40 $$\begin{array}{c}\dot{I}\left(t\right)=\left(B-\gamma I\right)I\left(t\right),\end{array}$$

where

$$B=\left[\begin{array}{ccc}{C}_{11}{p}_{11}{N}_{1}\left(0\right)/{N}_{1}\left(0\right)& \dots & {C}_{1n}{p}_{1n}{N}_{1}\left(0\right)/{N}_{n}\left(0\right)\\ \vdots & \ddots & \vdots \\ {C}_{n1}{p}_{n1}{N}_{n}\left(0\right)/{N}_{1}\left(0\right)& \dots & {C}_{nn}{p}_{nn}{N}_{n}\left(0\right)/{N}_{n}\left(0\right)\end{array}\right].$$

The eigenvalue of matrix $B-\gamma I$ determines whether the infectious disease will spread or not.

Since *B* represents individuals’ contact patterns, it is unlikely that one age group has no contact with another age group at all. Therefore, assuming that all elements of *B* are positive, the Perron–Frobenius theorem states that the eigenvalue of *B* with the largest absolute value, ${\beta }_{0}$ (Perron–Frobenius root), is the single root and is a positive real number.^27^ At the same time, it is the one with the largest real part among the eigenvalues of *B*. Also, the eigenvalues of the matrix $B-\gamma I$ are $\beta -\gamma$; therefore, ${\beta }_{0}-\gamma$ is the largest real part of the eigenvalues of $B-\gamma I$.^28^ From Eq. (40), the infection will spread, if ${\beta }_{0}-\gamma >0$. ${\beta }_{0}/\gamma$ is defined as the basic reproduction number in the multidimensional SIR model, and if it is greater than 1, the epidemic is expanding. The eigenvector corresponding to the Perron–Frobenius root specifies the distribution of infectious individuals by the age group.^29^

In theory, ${p}_{ij}$ in the matrix *B* all can take on different values, but when *B* is identified from the actual data, we are forced to make one of the following assumptions that reduce the number of parameters:

1. They all have the same value (${p}_{ij}=p$).
2. The infectivity is the same, but the susceptibility to infection differs by age group (${p}_{ij}={p}_{j}$).
3. They have the same susceptibility to infection, but the infectivity differs by age group (${p}_{ij}={p}_{i}$).
4. The infectiousness and susceptibility to infectivity differ by age group (${p}_{ij}={p}_{i}{p}_{j}$).

POLYMOD is a famous large-scale project that studies contact patterns among social groups in several European countries. A similar study was conducted in Japan by Ibuka et al. (2016) and Munasinghe et al. (2019). Akbarpour et al. (2020) recently attempted constructing a more detailed contact matrix in US metropolitan areas.

An overall reduction in contact, such as “refraining from going out by 80%,” attempts to lower this element of the matrix uniformly. However, with such detailed data for each group, the effects of targeted contact reduction can be estimated.

### Targeted lockdown

Since policies that vary restrictions according to the type of individual allow for more flexibility, they are more efficient than those that impose uniform restrictions on economic activity without distinguishing between individual types. However, if identifying individual types involves an enormous cost, uniform restrictions may be more cost-effective, so uniform restrictions are not necessarily denied. Such reasoning is what we saw in the discussion on uniform testing in Sect. 6.

However, there are social groups that can be identified without cost, with significant differences in the effectiveness of policies. There is literally an order of magnitude difference between the elderly and the young in terms of the fatality rate of COVID-19, as shown in Ferguson (2020) and the United States Center for Disease Control (CDC)’s modeling scenario.^30^ In terms of industry, there is also a difference between industries with high and low risk of infection. Using a multidimensional SIR model, we must consider policies that set different restrictions on economic activities for different social classes with different risks.

In the previous discussion, we have set the utility of a representative individual as the policy objective, but we face conceptual difficulties in identifying the social welfare function in a multidimensional SIR model. Glover et al. (2020) specified a social welfare function, whereas Acemoglu et al. (2020a) did not set a social welfare function but a monetized welfare loss like (19).^31^ The present paper follows the latter approach and employs a setting that is consistent with Sect. 5. The optimization problem becomes

41 $$\begin{array}{*{20}c} {\mathop {\min }\limits_{{y_{i} \left( t \right)}} \mathop \int \limits_{0}^{\infty } {\text{e}}^{{ - \left( {\rho + \nu } \right)t}} \left[ {\mathop \sum \limits_{i} N_{i} \left( t \right)y_{i} \left( t \right)\overline{Y} + \mathop \sum \limits_{i} \phi_{i} \left( {\mathop \sum \limits_{j} I_{j} \left( t \right)} \right)\gamma I_{i} \left( t \right){\text{VSL}}_{i} } \right]{\text{d}}t,} \\ \end{array}$$

where ${y}_{i}$ is a restraint on age group *i*’’s activity.^32^ The dynamics of new infections, which is a counterpart of (5) in a uni-dimensional model, is

42 $$\begin{array}{c}{\dot{I}}_{i}\left(t\right)={\mathrm{New}}_{i}\left(1-y\left(t\right),I\left(t\right),R\left(t\right)\right)-\gamma {I}_{i}\left(t\right),\end{array}$$

where $1-y$ is the vector representing restraint on each age group’s activity and *R* is the vector representing the removed in each age group. The dynamics of removed of each age group is

43 $$\begin{array}{c}{\dot{R}}_{i}\left(t\right)=\gamma {I}_{i}\left(t\right),\end{array}$$

and the number of deaths by the age group is given by

44 $$\begin{array}{*{20}c} { - \dot{N}_{i} \left( t \right) = \phi_{i} \left( {\mathop \sum \limits_{j} I_{j} \left( t \right)} \right)\gamma I_{i} \left( t \right).} \\ \end{array}$$

The optimization problem is to solve (41) subject to (42), (43) and (44). Let the Hamiltonian be

$${H}^{*}\left(t\right)={\mathrm{e}}^{-\left(\rho +\nu \right)t}H\left(t\right)={\mathrm{e}}^{-\left(\rho +\nu \right)t}\left\{\sum_{i}{N}_{i}\left(t\right){y}_{i}\left(t\right)\overline{Y }+\sum_{i}{\phi }_{i}\left(\sum_{j}{I}_{j}\left(t\right)\right)\gamma {I}_{i}\left(t\right){\mathrm{VSL}}_{i}+\sum_{i}{\lambda }_{I,i}\left(t\right)\left[{\mathrm{New}}_{i}\left(1-y\left(t\right),I\left(t\right),R\left(t\right)\right)-\gamma {I}_{i}\left(t\right)\right]+\sum_{i}{\lambda }_{R,i}\left(t\right)\gamma {I}_{i}\left(t\right)+\sum_{i}{\lambda }_{N,i}\left(t\right)\left[-{\phi }_{i}\left(\sum_{j}{I}_{j}\left(t\right)\right)\gamma {I}_{i}\left(t\right)\right]\right\}.$$

If the optimal restraint on each age group’s activity is interior, the first-order condition for age group *i* is given by

45 $$\begin{array}{*{20}c} {\frac{\partial H\left( t \right)}{{\partial y_{i} \left( t \right)}} = N_{i} \left( t \right)\overline{Y} - \mathop \sum \limits_{j} \lambda_{I,j} \left( t \right)\frac{{\partial {\text{New}}_{j} \left( t \right)}}{{\partial \left( {1 - y_{i} \left( t \right)} \right)}} = 0.} \\ \end{array}$$

Rearranging (45) yields

46 $$\begin{array}{*{20}c} {\mathop \sum \limits_{j} \lambda_{I,j} \left( t \right)\frac{{\partial {\text{New}}_{j} \left( t \right)}}{{\partial \left( {1 - y_{i} \left( t \right)} \right)}} = N_{i} \left( t \right)\overline{Y},} \\ \end{array}$$

which shows the property of optimal solution more clearly. Which age group will be more restricted can be considered by Eq. (46), when different levels of restrictions on behavior can be applied to each age group. The left-hand side is the benefit of reducing the number of deaths by restricting the behavior of a certain age group; the inclusion of ${\lambda }_{I,j}$ means that when comparing the benefit of reducing the number of infectious persons, reducing the number of infectious persons in the age group with the highest risk of death will be more beneficial. However, this does not immediately imply that the behavior of the age group at higher risk of death should be more strongly restricted. The benefit of restricting the behavior of other age groups to reduce the number of people infected by the age group at higher risk of death should also be considered. If this benefit is large, restricting the behavior of other age groups may be preferred. Also, strengthening restrictions on age groups with smaller populations may be recommended because costs are proportional to the population of an age group.

Let us specify the model and examine the effect of the ratio of age groups to the population. Consider an age group consisting of two types of people: the young (denoted by subscript 1) and the elderly (subscript 2). The fatality rate of the young is assumed to be approximated by zero, and the social costs of the young’s infection is ignored (${\lambda }_{I,1}=0$). Under the density-dependent contact, the new infectious is given by

47 $$\begin{array}{c}\begin{array}{c}{\mathrm{New}}_{i}\left(t\right)={\beta }_{i1}\left(t\right)\left(1-{y}_{i}\left(t\right)\right){S}_{i}\left(t\right)\left(1-{y}_{1}\left(t\right)\right){I}_{1}\left(t\right)\\ +{\beta }_{i2}\left(t\right)\left(1-{y}_{i}\left(t\right)\right){S}_{i}\left(t\right)\left(1-{y}_{2}\left(t\right)\right){I}_{2}\left(t\right).\end{array}\end{array}$$

If initial restraint does not exist, the ratio of marginal benefit to marginal cost of restraint on the young is calculated from (47) and is given by

$$\frac{{\lambda }_{I,2}{\beta }_{21}{S}_{2}{I}_{1}}{{N}_{1}\overline{Y} }.$$

By similar calculation, the ratio of marginal benefit to marginal cost of restraint on the elderly is given by

$$\frac{{\lambda }_{I,2}\left({\beta }_{21}{S}_{2}{I}_{1}+2{\beta }_{22}{S}_{2}{I}_{2}\right)}{{N}_{2}\overline{Y} }.$$

Since only the social costs of the elderly’s infection are considered, the marginal benefits are greater for imposing restrictions on the behavior of the elderly. Moreover, since the young population is usually larger than that of the elderly, a lower marginal cost is expected for the elderly. The model here assumes that the productivity of the young and the elderly is equal. If the contribution of the retired elderly to production is lower, this could be a further reason for the lower marginal cost of the elderly. These reasons support the activity restrictions on the elderly.

Acemoglu et al. (2020a) analyzed policies that impose activity restrictions on each of the three age groups (20–49, 50–64, and 65 years and above) and found that policies that impose heavier restrictions on the elderly than on the young are desirable. Their simulation shows that the simplistic policy of protecting the elderly and imposing almost no restrictions on the activities of the young is almost identical to the consequences of the detailed policy, indicating the importance of distinguishing between the elderly and the rest.

If contact between the young and the elderly and that within the young can be restricted separately, even more effective policies can be created. However, this does not mean that such policies can be implemented in the real world. The strategy of isolating the elderly with almost no restrictions on the activities of the younger generation is similar to the measures taken in Sweden, but in reality, the mortality rate of the institutionalized elderly became high, thus implying they were not well protected from the outbreak outside the institutions. In reality, we should consider the danger that we cannot reduce the risk of infection among the elderly as easily as in the model.

With targeted restriction, there is a disparity in burden between those who are restricted and those who are not. The welfare loss presented in Eq. (41) evaluates the total burden and does not consider the dispersion of the burdens, because the burdens are implicitly assumed to be equalized among individuals through income redistribution. The absence of compensation for those who are restricted may increase the political and social tensions.

To implement the solution of the model in the real world, more detailed models need to be analyzed. For example, if restrictions are to be imposed by industry, different model formulations will be needed.^33^

### Lockdown by industry and reopening scenario

Baqaee et al. (2021) examined the question of which industries should be given priority at the stage of lifting lockdown (reopening the economy). They used a multidimensional SIR model that divides the population into five age groups (0–19, 20–44, 45–64, 65–74, and 75 years and above). The model also includes incubation period, isolation, and death status. The number of contacts is set based on POLYMOD data, and the location of contact is distinguished into three categories: home, workplace, and other. The contacts of age group *i* with age group *j* is represented as the sum of contacts in the three locations:

48 $$\begin{array}{*{20}c} {C_{ij} \left( t \right) = C_{ij}^{{{\text{home}}}} \left( t \right) + \mathop \sum \limits_{k} C_{ij,k}^{{{\text{work}}}} \left( t \right) + C_{ij}^{{{\text{others}}}} \left( t \right),} \\ \end{array}$$

where the contacts at workplace are classified by 66 industries (indexed by *k*). The contacts at a particular industry’s workplace are assumed to be proportional to the number of workers there (${C}_{ij,k}^{\mathrm{work}}\propto {L}_{i,k}$).

The workers by the age group and by industry are obtained from American Community Survey. The output of each industry is assumed to be an increasing function of workers, and is written as

$${Y}_{k}\left(t\right)={Y}_{k}\left(\sum_{i}{L}_{i,k}\left(t\right)\right)={Y}_{k}\left({L}_{k}\left(t\right)\right).$$

The aggregate real output and each industry’s real output can be related by

49 $$\begin{array}{*{20}c} {P\left( t \right)Y\left( t \right)N\left( t \right) = \mathop \sum \limits_{k} P_{k} \left( t \right)Y_{k} \left( {L_{k} \left( t \right)} \right),} \\ \end{array}$$

where *P* and ${P}_{k}$ are a price deflator. Totally differentiating (49) and dividing it by nominal output yield

50 $$\begin{array}{*{20}c} {\frac{{{\text{d}}\left( {Y\left( t \right)N\left( t \right)} \right)}}{Y\left( t \right)N\left( t \right)} = \mathop \sum \limits_{k} \frac{{P_{k} \left( t \right)}}{P\left( t \right)Y\left( t \right)N\left( t \right)}\frac{{\partial Y_{k} }}{{\partial L_{k} }}\left( t \right){\text{d}}L_{k} \left( t \right) = \mathop \sum \limits_{k} \frac{{w_{k} \left( t \right)L_{k} \left( t \right)}}{P\left( t \right)Y\left( t \right)N\left( t \right)}\frac{{{\text{d}}L_{k} \left( t \right)}}{{L_{k} \left( t \right)}}.} \\ \end{array}$$

The last equation of (50) is obtained using that marginal value product of labor is equal to wage *w* (${P}_{k}\partial {Y}_{k}/\partial {L}_{k}={w}_{k}$). Assuming only the number of workers at industry *k* and rearranging (50) yield

$$\frac{\mathrm{dlog}\left(Y\left(t\right)N\left(t\right)\right)}{\mathrm{d}{L}_{k}\left(t\right)}=\frac{{w}_{k}\left(t\right){L}_{k}\left(t\right)/P\left(t\right)Y\left(t\right)N\left(t\right)}{{L}_{k}\left(t\right)},$$

which implies that the response of aggregate output to the number of workers in an industry is the ratio of the share of that industry’s labor income in nominal aggregate output to the that industry’s number of workers. In this way, the relationship between transmission occasions and economic activity by industry is quantitatively related.

However, if we use this model to determine which industries to reopen first, the timing of all industries becomes policy variables, and the number of policy variables is too large to be solved rigorously. Therefore, let us shelve the determination of the overall timing and consider how to weaken activity restraints for each industry to maximize production given the number of new infections at a certain time. Consider an increase in production of some industry, and calculate the ratio of the response of aggregate output to that of the basic reproduction number, at the initial point^34^:

51 $$\begin{array}{c}{\theta }_{k}=\frac{\mathrm{dlog}Y\left(0\right)N\left(0\right)/\mathrm{d}{L}_{k}\left(0\right)}{\mathrm{d}{\mathcal{R}}_{0}/\mathrm{d}{L}_{k}}.\end{array}$$

Baqaee et al. (2021) proposed a heuristic plan that reopen industries in decreasing order of this ratio and calculated this ratio by NAICS.^35^

The industries with the highest economic value per infection risk include finance, legal services, business management and administration, software development, and publishing. The high value-added of professional and technical services and the ability to work remotely are thought to be some reasons why these industries rank high. Since these industries have a strong presence in the US, but weak in Japan, whether these results apply directly to Japan is unclear.

Meanwhile, the industries with the lowest economic value per infection risk include health care, welfare, transportation, and education. They cannot be stopped or the cost of stopping them is high. However, the analytical framework here does not fully consider the cost of closing these industries. Other industries include food and beverage, accommodation, and entertainment, which have been the most heavily restricted industries.

The relationship between infection risks and productive activities could be improved in future studies. Although actual restrictions were implemented based on intuitive guesses in the absence of such study, the actual choice of industries is not significantly contrary to these estimates, and many of them seem to have been reasonable. Since choosing industries and imposing restrictions will place heavy burden on the restrained industries, supporting these industries is extremely important.

## Conclusion

This paper has described recent findings on the normative analysis of combined private and governmental countermeasures against infectious diseases, in the context of COVID-19 epidemic. Based on a model that relates the economic activity to infectious disease epidemics, considering policies that maximize the social welfare provides a theoretical basis for designing countermeasures that take health and the economy into account simultaneously. Restricting the economic activity also suppresses the outbreak of infectious diseases, and therein lies the trade-off between health and economy. During the first wave of the epidemic, lockdown in many countries was a policy of restricting economic activities on a wide scale, and the economic damage was extremely large.

The efficiency may be increased by taking measures to restrict activities by selecting targets compared to uniform restriction of activities. Under each measure, there is a trade-off between health and economy, in the sense that economic damage is created when trying to reduce fatality loss, but compared with uniform activity restrictions, both fatality and economic losses may be reduced. Meanwhile, uniform activity restrictions and selective activity restrictions have no trade-off.

Some attributes for selecting targets can be identified with little or no cost, such as age and industry, and others cannot be identified without cost, such as close contact with infectious individuals and the presence of pathogen carriage. In the early stages of an infectious disease epidemic, uniform restrictions, such as lockdown, were practical because of the absence of the accumulated knowledge of the relationship between activity and infection risk, disaggregated by age group or industry, and limited testing capacity. In the second wave and beyond, countermeasures based on selective activity restrictions are feasible, and lockdown or looser forms of uniform restrictions on economic activity are unlikely to be used. The search for desirable measures does not follow a health–economic trade-off between the measures implemented so far, but rather the possibility of improving both.

This paper is only a partial review of the large amount of research on the normative analysis, and some many important issues and challenges are not discussed in detail. Finally, we close this paper by pointing out four challenges. First, knowledge of the parameters of the models underlying normative analysis is important, and more research is needed to obtain robust estimates. Even after the data from the experiences are available, the ambiguity of parameter leads to inconclusive evaluations. Particularly, the setting of the value of a statistical life for converting effects into benefits has a wide range and is also likely to overestimate the benefits. Second, knowledge of the effectiveness of targeted countermeasures needs to be deepened to identify more cost-effective countermeasures against infectious diseases. Third, the damage caused by infectious diseases and infectious disease control is unevenly distributed among individuals and classes. Implementing a mechanism of income redistribution that will equalize it across society as a real policy is not an easy problem. Fourth, while many studies are based on the SIR model, in which immunity persists, analyses based on the SIS model and other models, in which immunity does not persist, will also become important in the future.

## Data Availability

Not applicable.

## Appendix

See Table 3.

**Table 3:** Estimates of other parameters in ex post evaluations of lockdown

	Coverage of costs	Coverage of effects	Coverage is consistent	Costs per week	Costs	Costs are related with effects	Effects	Consideration of other effects
(United States)								
Broughel and Kotrous	Stay-at-home orders	Total	No	*35.7b*	*214.2b–331.5b*	No	940,000–1,040,000	Yes
Doti	Stay-at-home orders	Stay-at-home orders	Yes		410b	Yes	358,000	No
Scherbina	Stay-at-home orders	Total	No	36.93b	*147.72b*	No	*203,000*	Yes
Thunstrom et al.	Total	Total	Yes		7.21t	No	1,239,000	No
(United Kingdom)								
Miles et al.	Total	Total	Yes		(9–25% of GDP)	No	20,000–440,000	No
Rowthorn and Maciejowski	Total	Total	Yes	200 (per capital)		Yes	380,000	Yes

This table presents the estimates of parameters that did not appear in Table 2. The unit of costs is US dollars and UK pounds. Italic indicates the value not reported in the reference, but complemented by the author. “Coverage is consistent”: when costs and effects are estimated, the study focuses on the same measures against COVID-19. “Costs are related with effects”: the study explicitly connects the effects of measures against COVID-19 with their costs. “Effects”: lives saved. “Consideration of other effects”: the effects include not only lives saved but also reduction of non-fatal loss.

## Footnotes

1 Since this paper focuses on COVID-19, only human-to-human transmissions are considered.

2 Using French weekly data on flu-like illness, acute diarrhea, and chickenpox, Adda (2016) found that the diffusion of diseases is pro-cyclical.

3 Since this paper focuses mainly on normative analysis, many positive analyses are outside the scope of this paper. Complementary reviews which cover them include the works of Avery et al. (2020) and McAdams (2021). McAdams (2021) explained developments of economic epidemiology prior COVID-19, and Avery et al. (2020) covered the studies which appeared just after the emergence of COVID-19.

4 Some preceding studies incorporated economic aspects into the SIR model. For instance, Gersovitz and Hammer (2004) developed the analytical framework of the dynamic optimization problem in the SIR model by incorporating the costs of lowering the transmission rate. In the SIR model, Philipson (1995) takes account of the incentive to be vaccinated.

5 It is called the “density-dependent,” because the number of contacts is proportional to the population per area. The mass action of air in the given volume can be described by this specification.

6 Garibaldi et al. (2020a) described the relationship between search theory and SIR model. Petrongolo and Pissarides (2001) reviewed the research on matching function.

7 Ellison (2020) extensively discussed the introduction of heterogeneity into SIR models.

8 As explained by Avery et al. (2020), epidemiology employs the two approaches to explaining the relationship between behavior and infection in SIR models. The “mechanical” approach, like a structural form of economic model, is oriented toward describing the mechanisms of infection in detail, providing a mechanism of policy effects for the analysis of countermeasures to infectious disease. Meanwhile, the “phenomenal” approach attempts to describe the epidemic without placing too many constraints on the model when making predictions (e.g., Atkeson et al., 2020; Fernandez-Villaverde & Jones 2020). Both approaches have advantages, depending on the purpose for which the model is used.

9 This simplifying assumption misses that in the COVID-19 outbreak, a sharp drop in income concentrated on vulnerable workers, thus creating a gap between their income and consumption. Although massive fiscal supports aimed to offset their disadvantageous status, it is outside the scope of this paper.

10 For example, Alvarez et al. (2020).

11 Farboodi et al. (2021) and Garibaldi et al. (2020a) discussed this point.

12 Note that ${\lambda }_{N}\left(0\right)$ is different from ${\lambda }_{N,0}$, which is the lifetime utility in the pre-epidemic economy.

13 Since the surviving probability is initially 1, its increase is unrealistic. A reasonable situation is the case of small decrease in surviving probability from 1.

14 Rowthorn and Toxvaerd (2012) and Toxvaerd (2020) considered the model that has a corner solution.

15 In Gersovitz and Hammer’s (2004) pioneering work of considering the economic incentives of prevention measures in the SIR model, they missed the dynamic externalities, because they employed the setting of large household that takes into account the effects of their behavior on the aggregate state variables (the large household internalizes the dynamic externalities). Meanwhile, Garibaldi et al. (2020b), Gersovitz and Hammer (2004) and Gonzalez-Eiras and Niepelt (2020b) explicitly modeled private sector’s optimization problem and characterized the static externality as a difference in perception of the efficacy of prevention behavior. In the model described here, $\partial New/\partial \left(1-y\right)$ in Eq. (25) is different between social planning and private optimization. Section 4.1 has given an intuitive example of static externality.

16 The following classification of dynamic externalities is drawn from Garibaldi et al. (2020b). Since they set a model differently, the exact matching of concepts is not guaranteed. Particularly, they employed *S* and *I* as state variables, whereas this paper uses *I* and *R*.

17 “Exposed” stands for those who are infected but stay asymptomatic.

18 Among them, Doti (2021) and Rowthorn and Maciejowski (2020) did not derive the net social benefit, which is a central concept of cost–benefit analysis.

19 This implication is derived from their original functional form, $y\left(t\right)=0.35{\left(\frac{x\left(t\right)}{0.75}\right)}^{3}$, where $x\left(t\right)\equiv \frac{{\beta }_{0}-\beta \left(t\right)}{{\beta }_{0}}$. 1.064 approximates $0.75/{0.35}^{1/3}$.

20 Looking at only the loss of market output overlooks the gain in home production, which is suspected to happen due to voluntary or mandatory staying at home. This ignorance may overestimate the cost of prevention measures.

21 Estimates of other parameters are presented in Table 3 of Appendix.

22 Kaplan et al. (2020) did not aggregate the consequences of heterogeneous agents, but simulated the distribution of economic costs and deaths under the lockdown policy that mimics the US experience in spring 2020. They found that lockdown burdens are uneven, thus implying that concentrating on the aggregates will miss an important social problem.

23 In actual testing, the probability of determining a positive person to be a positive person (sensitivity) and a negative person to be a negative person (specificity) is less than one, and in some cases, a positive person is mistakenly determined to be a negative person (false negative) or a negative person is mistakenly determined to be a positive person (false positive). The existence of false negatives and false positives, both of which have the effect of decreasing the efficiency of isolation, may be incorporated in the model. It should also be noted that the existence of false positives has the adverse effect of restricting their activities.

24 A similar analysis can be conducted under the alternative assumption that they will not be subject to testing.

25 Acemoglu et al. (2020b) pointed out that it may not always be desirable to increase the frequency of testing due to behavioral factors that are dismissed in this paper. The increase in testing frequency may increase the chance of infection by making tested negatives more active.

26 Isolation restricts the behavior of those whose infection is confirmed, whereas quarantine restricts the behavior of those who are suspected to be infected.

27 ${\beta }_{0}$ is a positive real number, and has the largest absolute value among eigenvalues. Therefore, no other eigenvalue has a larger real part.

28 Define the eigenvalue of matrix *B* by *β*. Since $\left|B-\beta I\right|=0,\space\left|B-\gamma I-\left(\beta -\gamma \right)I\right|=0$. Therefore, the eigenvalue of matrix $B-\gamma I\,\mathrm{is}\,\beta -\gamma$. Since ${\beta }_{0}$ has the largest real part among *β*, the eigenvalues of matrix *B*, ${{\beta}_{0}}-{\gamma}$, has the largest real part among the eigenvalues of $B-\gamma I$.

29 Let *x* be the eigenvector for β. Since $Bx=\beta x$, $\left(B-\gamma I\right)x=\left(\beta -\gamma \right)x$. Thus, the eigenvector of β is also the eigenvector for the eigenvalue $\beta -\gamma$ of the matrix $B-\gamma I$. All elements of the eigenvector for Perron–Frobenius root are positive. It guarantees that the number of infectious is positive.

30 COVID-19 Pandemic Planning Scenarios. https://www.cdc.gov/coronavirus/2019-ncov/hcp/planning-scenarios.html.

31 If equivalent variations are aggregated over heterogeneous individuals, the evaluation of VSL will bring a controversial, ethical issue. Since the willingness to pay for a particular good usually increases with income, this procedure is obliged to imply that saving rich people is socially more valuable. Thus the practice of cost–benefit analysis rejects to vary the VSL among income groups. Adler et al. (2014) discussed another approach to employing a social welfare function that offsets this troublesome income effect on VSL.

32 If an older group is priotized in vaccination, the discount rate may vary among groups. Which group is given the priority is an important policy question, and it should be analyzed as an optimization problem, where each group’s *v* is a policy variable.

33 Based on Italian experiences, Favero et al. (2020) considered targeted lockdown by the age group and by production sector. Since their two-production-sector setting leads to a limited implication for planning a targeted lockdown by industry, some studies have called for a detailed industrial classification.

34 The ratio can be evaluated at the point of policy enactment, not at the initial point. In this case, the right-hand side of (51) should take the effective reproduction number instead of the basic reproduction number.

35 Differentiation in the denominator of the right-hand side of (51) is defined by assuming that the largest real part of eigenvalue of the matrix in (40) is a function of elements of the matrix. In turn, we assume that the proportional relationship between contacts and workers shown by (48) holds in the pre-epidemic economy; therefore, the elements of matrix are a function of the number of workers in an industry.
